# External Force Field for Protein Folding in Chaperonins—Potential Application in *In Silico* Protein Folding

**DOI:** 10.1021/acsomega.4c00409

**Published:** 2024-04-10

**Authors:** Irena Roterman, Katarzyna Stapor, Dawid Dułak, Leszek Konieczny

**Affiliations:** † Department of Bioinformatics and Telemedicine, Jagiellonian University—Medical College, Medyczna 7, Kraków 30-688, Poland; ‡ Faculty of Automatic, Electronics and Computer Science, Department of Applied Informatics, Silesian University of Technology, Akademicka 16, Gliwice 44-100, Poland; § ABB Business Services Sp. z o.o, ul Żegańska 1, Warszawa 04-713, Poland; ∥ Chair of Medical Biochemistry—Jagiellonian University—Medical College, Kopernika 7, Kraków 31-034, Poland

## Abstract

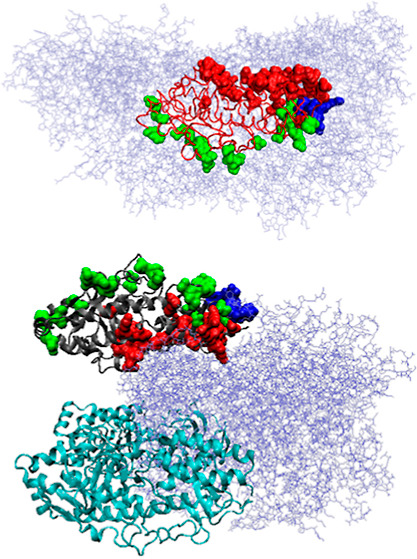

The present study discusses the influence of the TRiC chaperonin involved in the folding of the component of reovirus mu1/σ3. The TRiC chaperone is treated as a provider of a specific external force field in the fuzzy oil drop model during the structural formation of a target folded protein. The model also determines the status of the final product, which represents the structure directed by an external force field in the form of a chaperonin. This can be used for *in silico* folding as the process is environment-dependent. The application of the model enables the quantitative assessment of the folding dependence of an external force field, which appears to have universal application.

## 1 Introduction

Proteome is the complete set of proteins that represent all biological functions in a given organism.^1^ The proteome includes proteins with a broad spectrum of biological activities (*e.g.*, enzymes or receptors) as well as structural proteins. One of the groups of proteins that guard protein homeostasis is chaperones, which contain chaperonins as a subgroup.^2^ The role of these proteins is to control the correct protein folding including the correction of misfolded proteins. Studies aimed at understanding the importance of the chaperonin family have focused on bacterial (*Escherichia coli*) GroEL. These proteins can be referred to as machines because of their large size, complexity of the process in which they are involved, and the need for ATP to support their activity. Large complexes comprise numerous chains (often >10) that form a dual-ringed tetradecamer with the *CN* axis of symmetry, where the *C*-axis symmetry is the axis that passes through the axis of the capsid, and *N* denotes the number of chains in one-half of the capsid. These proteins can also participate in transport as done by prefoldin.^3^ GroEL promotes folding inside the capsid. This process requires energy (ATP) and proceeds with a temporarily significant change in the ordered symmetrical structure of GroEL. The arrangement of chambers into which the inserted polypeptide chain undergoes conformational changes ensures that the folding protein structure maintains its function.^4^

The possibility of incorrect folding increases with temperature, with a large amount of incorrectly folded proteins appearing under elevated temperatures, along with subsequent incorrect complexation. These proteins are often referred to as heat-shock proteins.^5^ The chaperone group also includes small-sized proteins that form transient complexes during the folding process, thus preventing the early complexation of sections of a synthesized protein before the appropriate complexation that produces the correct tertiary structure.^6–11^

Herein, we focused on the chaperonin TRiC in complex with an aggregation-prone protein subunit of a viral capsid (PDB ID: 7LUP(12)). The substrate for the chaperonin in question is the reovirus σ3 capsid protein, especially the σ3 chain of the mu1/σ3 complex. The structure of this chain is also available in a final biologically active reovirus mu1/σ3 complex (PDB ID: 1JMU(13)), which enables a comparative analysis of the structure of this chain, with its form present in TRiC. The detailed analysis is limited to the σ3 chain of the virus–capsid complex. The present study treats the GroEL chamber system as a local external force field that provides external conditions under which the structure of a biologically active protein can be folded in this environment. The chains of GroEL in immediate contact with the target molecule are discussed.

## 2 Materials and Methods

### 2.1 Fuzzy Oil Drop Model

This study applied the fuzzy oil drop (FOD) model.^14,15^ A short description is presented here to facilitate the interpretation of the results obtained by using the model. The basic assumption introduces treating a protein structure as an effect of the micellization process. Amino acids are bipolar molecules with a diverse polarity–hydrophobicity relationship. Bipolar molecules in an aqueous environment form ordered arrangements with a distribution characterized by a hydrophilic surface, with hydrophobic residues concentrated in the center. The hydrophobicity distribution in such a system can be described by a three-dimensional (3D) Gaussian function spanning the protein molecule. The values of the parameters (σ_*X*_, σ_*Y*_, and σ_*Z*_; eq 1) are adopted to the size and shape of the molecule, thereby allowing the representation of any globular form of the protein molecule.
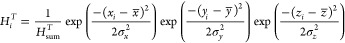
1

**Figure 1:**
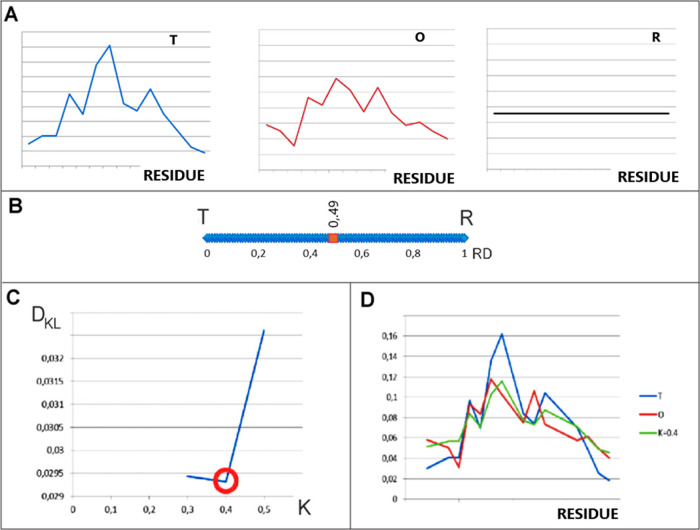
Graphical presentation of the FOD-M model. (A) *T*, *O*, and *R* distribution for the selected example object. (B) The determined RD value (RD < 0.5) for this example indicates the presence of a hydrophobic core despite visible differences. (C) Determination of the value of the *K* parameter was based on the search for the minimum *D*_KL_ value for the *O*|*M* relationship. (D) Juxtaposition of the *T*, *O*, and *M* profiles for the *K* value [as in (C)]. Vertical axes in (A,D) represent hydrophobicity.

The value of the 3D Gaussian function at points representing consecutive amino acids (*i.e.*, positions of the effective atoms, which are the averaged positions of the atoms comprising a given amino acid) expresses the idealistic hydrophobicity level referred to in the model as the theoretical *T*_i_ (Figure 1A).

In exceptional cases, the structure of the protein exactly reproduces a distribution according to a 3D Gaussian function. The actual hydrophobicity level of a given amino acid results from the intrinsic hydrophobicity of each amino acid and its interaction with its neighbors (*r*_*ij*_: distance between effective atoms). The Levitt function was used for the calculation of the observed hydrophobicity level (*O*)^16^ (eq 2; Figure 1A).
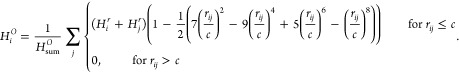
2

The *T* and *O* distributions after normalization can be subjected to comparative analysis.

The normalization is expressed by 1/*H*_sum_^*T*^ for the *T* distribution and 1/*H*_sum_^*O*^ for the *O* distribution. The *T*_i_ and *O*_i_ are calculated for all residues. To make them normalized, each of the compounds is divided by the sum of all *T*_ij_ and *O*_ij_. Quantitatively, the compatibility/incompatibility of the *O* distribution against the *T* distribution (reference distribution) is expressed by divergence entropy introduced by Kullback–Leibler^17^ (eq 3).
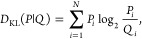
3where *P*_*i*_ is the distribution analyzed (*O* distribution in our model) and *Q*_*i*_ is the reference distribution (*T* in the FOD model).

However, the *D*_KL_ value (entropy) cannot be interpreted. Therefore, another reference distribution (*R*) (Figure 1A) was introduced in which each amino acid represents the same level of hydrophobicity equal to 1/*N*, where *N* is the number of amino acids in the structural unit under consideration.

The *D*_KL_ value is determined for the relationship of the *O* distribution toward the *R* distribution (the *O*|*R* relationship). A comparison of these two *D*_KL_ values indicates the similarity of the *O* distribution to one of the two reference distributions (*T* and *R*). A smaller *D*_KL_ value indicates the similarity of the compared distributions. The relative distance (RD) value of a protein is expressed as follows
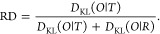
4where RD < 0.5 is interpreted as a protein with a hydrophobic core (Figure 1B).

A protein composed of amino acids linked by covalent bonds (peptide bonds) has limited possibilities (limited mobility) to reproduce the micelle structure. The degree of adaptation of the *O* distribution toward the *T* distribution appears to vary. Down-hill, fast-folding, ultrafast-folding proteins represent a status with very low RD values;^18^ enzymes, whose structure requires a substrate-binding cavity, show a local mismatch in the form of local hydrophobicity;^19–21^ the complexation area can be recognized as a local hydrophobicity excess.^22,23^ The elimination of residues of highest discrepancy between the *O*_i_ and *T*_i_ values enables the identification of a moiety fulfilling the condition of RD < 0.5, indicating that this is responsible for solubility. The local discrepancies may also be recognized as a potential drug-binding locus.^24^

**Figure 2:**
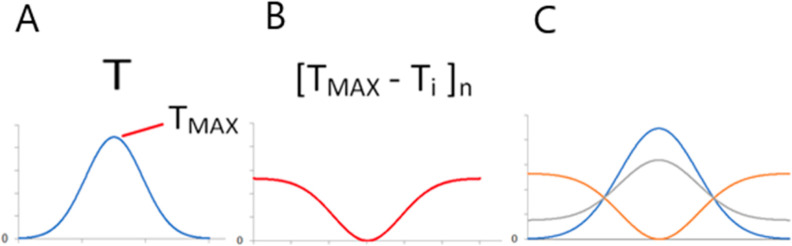
Steps in the *K* calculation: (A) Gauss function with *T*_MAX_ was indicated. (B) Opposite function calculated as [*T*_MAX_ – *T*_i_]_*n*_, where index *n* denotes normalization. (C) The *T* function (blue line) transformation to [*T*_MAX_ – *T*_i_]_*n*_ (red line) and *M* form for *K* = 0.4 show the relations between stepwise changes of the *T* function transformation.

Water is not the only milieu for protein activity. The exposure of hydrophobic residues on the surface is required for the stability of the membrane-anchored protein, which is opposite to the water environment. This opposite distribution of hydrophobicity in membrane proteins can be expressed as a function complementing to 1. In practice, the opposite distribution is determined as follows (Figure 2)
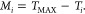
5

However, the omnipresence of water influences the form of the hydrophobic distribution in membrane proteins in the following form
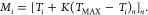
6

In the formula, the *K* parameter expresses the force with which the field originating from the water environment is modified by factors other than water, including hydrophobic factors (Figures 1C and 2).

The graphical presentation of opposite function and calculation of *K* parameter is shown in Figures 1D and 2.

The *M* distribution is calculated for the minimal value of *D*_KL_(*O*|*M*) (Figures 1C and 2) to find the modified *T* distribution, possibly the closest one with respect to the *O* distribution. The *M* distribution plays the role of *T* distribution in a nonaqueous environment modified by other factors. The description of the protein according to the final form of the FOD-modified (FOD-M) model is thus expressed in terms of the RD and *K* parameter values. The value of the RD parameter expresses the degree of similarity/dissimilarity of the *O* distribution to the *T* distribution (automatically the *R* distribution). The *K* parameter, however, expresses the extent to which the nonaqueous environment affects protein structure formation.

Proteins characterized by low *K* value s are listed in.^25,26^ A protein functioning in the periplasmic space exhibits a structure described by the parameter value *K* = 0.6.^27^ Membrane proteins (*e.g.*, rhodopsin described by the value *K* = 0.9) are described by *K* value s > 1.0.^28–32^ The present paper discusses a protein, chaperonin, which can represent a status with *K* > 3. A high value of the *K* parameter is also shown by a protein folded in the external field environment provided by chaperonin.

### 2.2 Pictorial Presentation of the FOD and FOD-M Models

**Figure 3:**
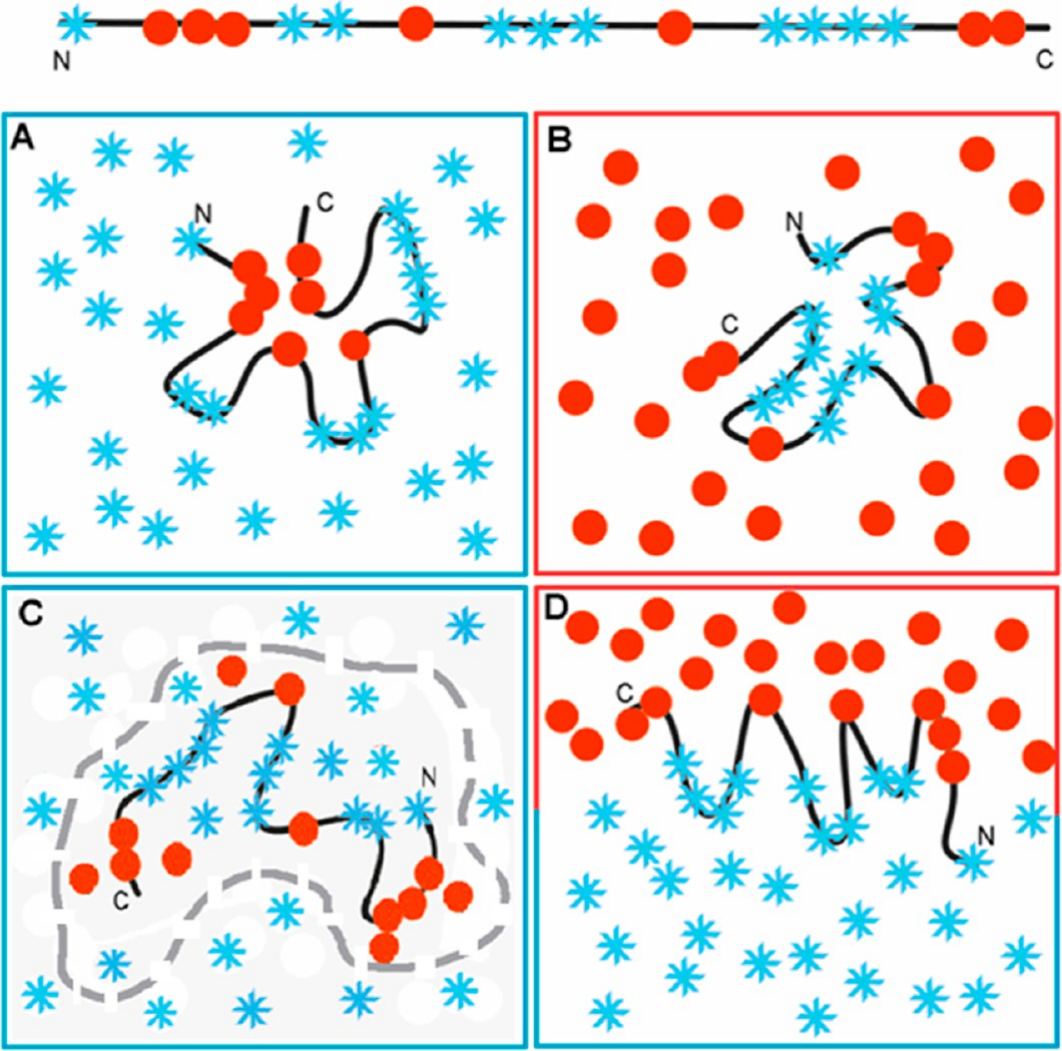
Structural folding of a polypeptide chain with hydrophobic and hydrophilic residues distributed as shown at the top (blue stars, hydrophilic residues; red dots, hydrophobic residues). (A) Folding in an aqueous environment, which directs the hydrophobic residues toward the central part, with hydrophilic residues exposed on the surface; hydrophobicity distribution expressed by 3D Gauss function (low RD and low *K* value s). (B) Folding in a hydrophobic (nonpolar) environment, concentrating the hydrophilic residues in the central part of the hypothetical protein. Such a structure is described by high RD and high *K* value s due to the absence of a hydrophobic core in the central part of the molecule. (C) Environment generated by chaperonin isolating the folding chain from the contact with water molecules as discussed in this paper. The gray line visualizes the chaperonin structure. The dashed line visualizes the much larger size of this molecule. The external environment for chaperonin is water (blue X). (D) Polarized environment with hydrophobic molecules in an associated form and distributed water molecules, producing an ordered structural form, but one which is far from the 3D Gauss hydrophobicity distribution, also having high RD and *K* value s. The colors in frames represent the form of local environment.

The main idea basis for the FOD model can be presented in a graphic form (Figure 3).

The polar water molecules (blue stars) surround and direct the folding polypeptide chain toward the generation of a hydrophobic core to centrally concentrate hydrophobic residues with a polar surface (Figure 3A). This type of structure is described by low RD and low *K* value s. The hydrophobic environment directing the folding process toward the hypothetic hydrophilic core formation with a hydrophobic surface (Figure 3B) represents the membrane directing the folding of the proteins to expose the hydrophobic residues on the surface. The specific distribution on the internal surface of GroEL (Figure 3C) may direct a well-defined folding that is compatible with the polar/hydrophobic residue exposure in chaperonin. The biphase environment directs the folding process toward amphipathic construction (Figure 3D). The cases shown in Figure 3B–D represent the status of high RD and high *K* value s according to the FOD-M model.

### 2.3 Programs Used

Profiles and RD values can be calculated *via* two approaches: 1. the CodeOcean platform: https://codeocean.com/capsule/3084411/tree. Please contact the corresponding author for access. 2. The application, implemented in collaboration with the Sano Centre for Computational Medicine (https://sano.science) and running on resources contributed by ACC Cyfronet AGH (https://www.cyfronet.pl) in the framework of the PL-Grid Infrastructure (https://plgrid.pl), provides a web wrapper for the abovementioned computational component and is freely available at https://hphob.sano.science. VMD software was used to present the 3D structures.^33,34^

## 3 Results and Discussion

The aim of this work was to determine how chaperonin affects the folding process inside a polypeptide chain. Chaperonin is treated as a provider of an external force field with a specificity that eliminates the influence of water molecules on its standard structuralization. To standardize the notation, the following nomenclature is used:

- TRiC: chaperonin structure without a substrate, comprising chains A–P (chains A–R in PDB ID: 7LUP).
- TRiC + Q: chaperonin structure with a “guest” molecule (complete structure as available in PDB ID: 7LUP).
- TRiC-A, TRiC-B: single-chain components of the TRiC chaperonin (PDB ID: 7LUP-A).
- Chain Q: “guest” molecule, an σ3 chain, a component of a complex with TRiC (chain Q in 7LUP-Q).
- Chain G: an σ3 chain, a component of the reovirus mu1/σ3 complex (native form) (PDB ID: 1JMU).

Mu1, the structure of the complete reovirus mu1/σ3 complex (PDB ID: 1JMU), is an arrangement of six chains A–F with C3 symmetry, together with three σ3 chains (G, H, and I), of which chain G is the subject of analysis. Chains identified as Q and G represent the same protein in different complexes, and therefore, structures of these two forms can be compared.

The present work comprises the following:

1. A comparative analysis of the G and Q chains treated as individual structural units (3D Gaussian function spanning each chain individually);
2. An analysis of the structure of the Q chain as a component of TRiC + Q, with a 3D Gaussian function spanning the complex;
3. An analysis of the structure of the G chain as a component of the reovirus mu1/σ3 complex, with a3D Gaussian function spanning the complex;
4. An analysis of the structure of TRiC, with a 3D Gaussian function spanning the chaperonin complex (chains A–P) without the presence of folding protein (chain Q absent), treated as a description of the external force-field specificity.

Chain G, the component of the reovirus mu1/σ3, is studied in detail. However, the status of all components (chains) is given in the Supporting Information (Table S1).

### 3.1 Comparative Analysis of the G and Q Chains Treated as Individual Structural Units (3D Gaussian Function Spanning Each Chain Individually)

A comparative analysis of the structures of the Q and G chains was performed by treating them as individual structural units. However, these proteins cannot be analyzed without considering their immediate environment as each protein is a component of a complex. Therefore, the status of the chains in question is additionally assessed together with the remaining chains within the complex, treating a chain as part of a larger system and as a separate component of the complex. This means that in addition to determining the 3D Gaussian function for an individual chain (Q or G), the 3D Gaussian function is determined for the entire complex. The *T*, *O*, and *M* distributions obtained for all components of the complex are evaluated. The assessment of the status of a chain as a component of the complex involves removing fragments representing a given chain from the profiles describing the complete complex. The *T*_i_, *O*_i_, and *R*_i_ values of a selected fragment (in this case, a chain) are normalized, and the RD and *K* value s are determined independently. Values determined in this way allow an assessment of the contribution of a given chain to the common structure of the complex, including, for example, the participation in micelle-like ordering or as a disruptive factor for such an arrangement.

The structures of the chains in question (Q and G) show a high degree of similarity as shown by an rmsd of 0.75 with a TM-score of 0.99 and a sequence identity of 97% (results according to [RCSB: https://www.rcsb.org/alignment] rmsd calculation). The arrangement of the secondary structure is common in both the structures.

The status of both forms of chains expressed using parameters introduced in the FOD model (Table 1) defines the structures of the individual Q and G chains (3D Gauss function defined for each unit) as highly unsuitable for the requirements of this model. A high mismatch with such a distribution implies a different hydrophobicity distribution devoid of a hydrophobic core. High *K* value s suggest that the structure of this protein is obtained as an effect strongly directed by the high participation of factors other than the polar water environment, as interpreted by the FOD-M model. The values of these parameters for the chains in question were very high, indicating a hydrophobic distribution without a hydrophobic core and a weakly polar surface. Notably, these structures differ to a minimum extent and have highly similar RD and *K* parameter values, although the parameters for the final structure as part of the reovirus σ3 capsid were higher.

**Table 1:** RD and *K* Parameter Values for Determining the Status of Individual Chains Treated as Individual Structural Units; Q and G Chains Are Treated as Components of Complexes^a^

	chain Q TRiC complex (7LUP)		chain G reovirus mu1/σ3 complex (1JMU)	
	RD	*K*	RD	*K*
individual	0.690	1.1	0.719	1.3
in complex chain contribution in the distribution of hydrophobicity as part of the entire complex	0.554	0.6	0.778	1.4
complete complex: all chains together	0.760	4.0	0.792	1.6
complex without the Q and G chains, respectively	0.752 (A–P)	3.6	0.781 (A–F, H, I)	1.7
			0.769 (A–F)	1.5

a The complete complex and chains constitute “partners” in the complex, excluding the chain in question (TRiC and reovirus σ3 capsid, respectively), which are the absent Q and G chains, respectively.

The status of the chains in question treated as components of complexes reveals a higher alignment of the Q chain arrangement against the TRiC complex than that of the final form of the G chain in the reovirus σ3 capsid complex. Conversely, the G chain in the reovirus σ3 capsid complex structure introduces a significant disruption of hydrophobicity distribution within the micelle-like ordering. This means that the presence of the Q chain in the complex with TRiC introduces a local ordering somewhat similar to the 3D-Gaussian compatible arrangement of the hydrophobicity distribution, although this does not reach a state of compatibility with this distribution.

The analysis of the complete complexes reveals a far from micelle-like ordering of hydrophobicity for TRiC. This is due to the obvious mismatch, especially for the central region (where the 3D Gaussian function expects the maximum density of hydrophobicity), which is filled by several water molecules representing the ordering influenced by the neighboring chaperonins. The *K* = 4.0 value obtained for the GroEL system with a folding chain present inside is the highest value obtained for any protein (complex) in the analysis to date. This high value is due to the absence of the hydrophobic core expected in the central part of the construction.

Providing RD and *K* value s for the components of the respective complexes devoid of the analyzed chain is intended to determine the specificity of the external force field for each chain. This is important for determining the status of GroEL as a provider of the immediate environment that creates the environment for the folding chain. This surrounding external force field for the Q chain is significantly different from the micelle-like arrangement. Parameters obtained for TRiC describe this complex as an external force field in which the protein folds. Interpretation of these parameters means that the folding process orientation is different from that for water. The model applied here allows quantitative assessment of the environment specificity, which differentiates this applied model from others discussing the same problem.

**Figure 4:**
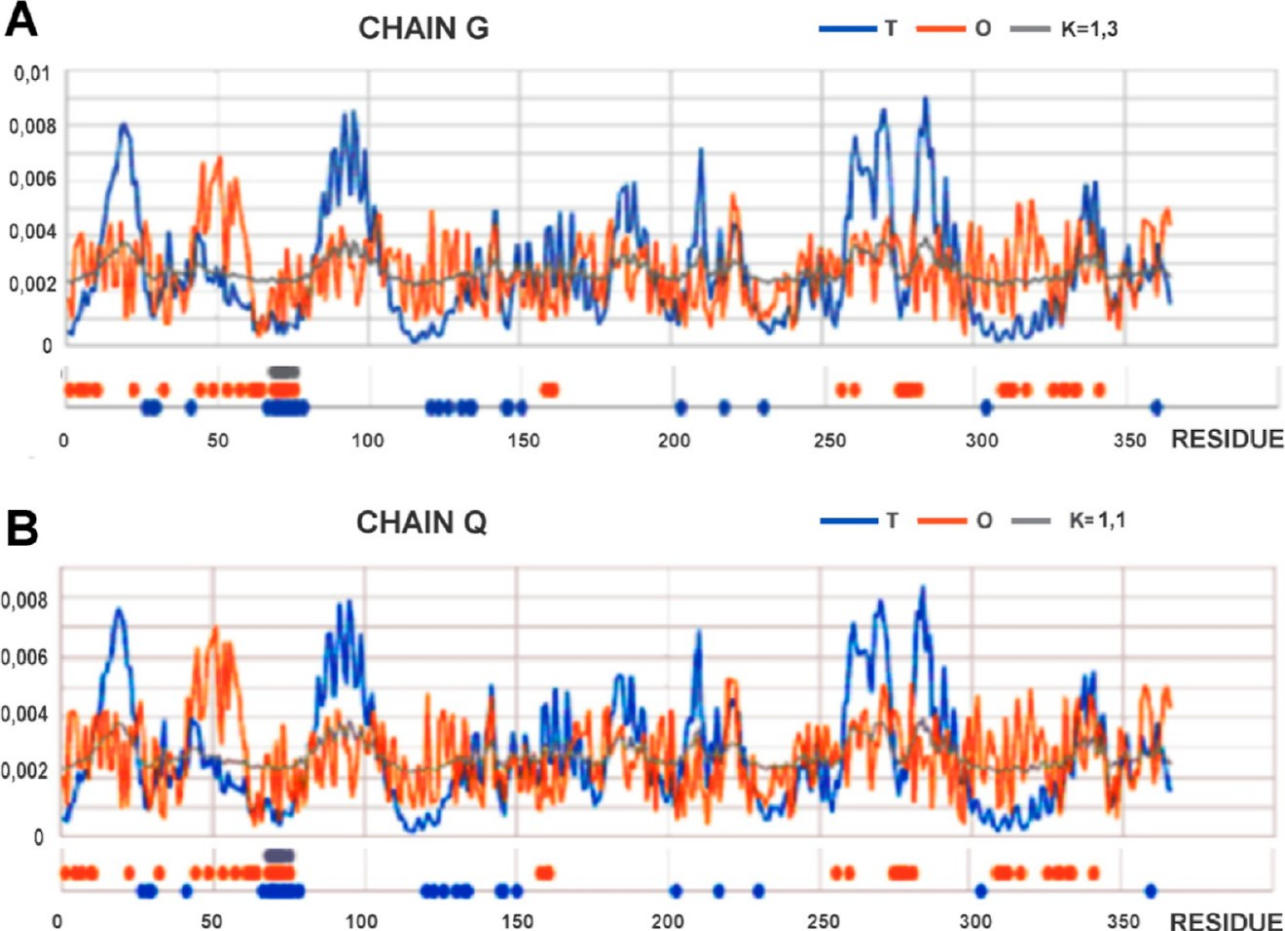
*T*, *O*, and *M* profiles for the Q/G chain (vertical axis: hydrophobicity). (A) G chain as an individual structural unit (extracted from the reovirus mu1/σ3 complex [PDB ID: 1JMU]). (B) Q chain treated as an individual structural unit (extracted from the TRiC/σ3 complex [PDB ID: 7LUP]). Horizontal axis positions: blue (bottom) represents residues involved in Q chain + TRiC complexation; red (central) represents residues involved in G chain + reovirus mu1/σ3 complex complexation, and gray represents residues involved in interactions in both complexes discussed.

The visualization of the *T*, *O*, and *M* profiles for the chains in question (Figure 4) reveals the specificity of their structuring. The structural similarity expressed by the rmsd value is also revealed in the shape of the *T* distribution profiles. This is because they show the center location (high *T*_i_ values) in the same positions, and the surface locations (low *T*_i_ values) are very similar in both cases. This was also true for the similarity of the *O* and *M* distributions.

The *M* distributions for chains treated as individual structural units show a degree of fitting with the *O* distributions in a limited number of regions, but they significantly diverge from the *T* distributions. These discrepancies were expressed both as a local deficit of hydrophobicity, especially in central regions that show high levels of *T*_i_ (with low *O*_i_ values), and as a local excess in parts located on the surface (low *T*_i_ with higher *O*_i_ values).

The reported positions of residues involved in the interface formation in both cases show different locations with several common positions (residues of the Q chain being in contact with both TRiC and the reovirus σ3 capsid for residues 68–75, gray line). This section shows a local excess of hydrophobicity on the surface. This may suggest the involvement of hydrophobic interaction in the structure of this local interface (gray line, Figure 4).

The isolated residues involved in the interaction with the TRiC chains indicate a much higher proportion of hydrophobic interactions as part of the interface than that in the reovirus mu1/σ3 complex. This observation is due to an increased level of hydrophobicity for residues interacting with TRiC (Figure 4).

**Figure 5:**
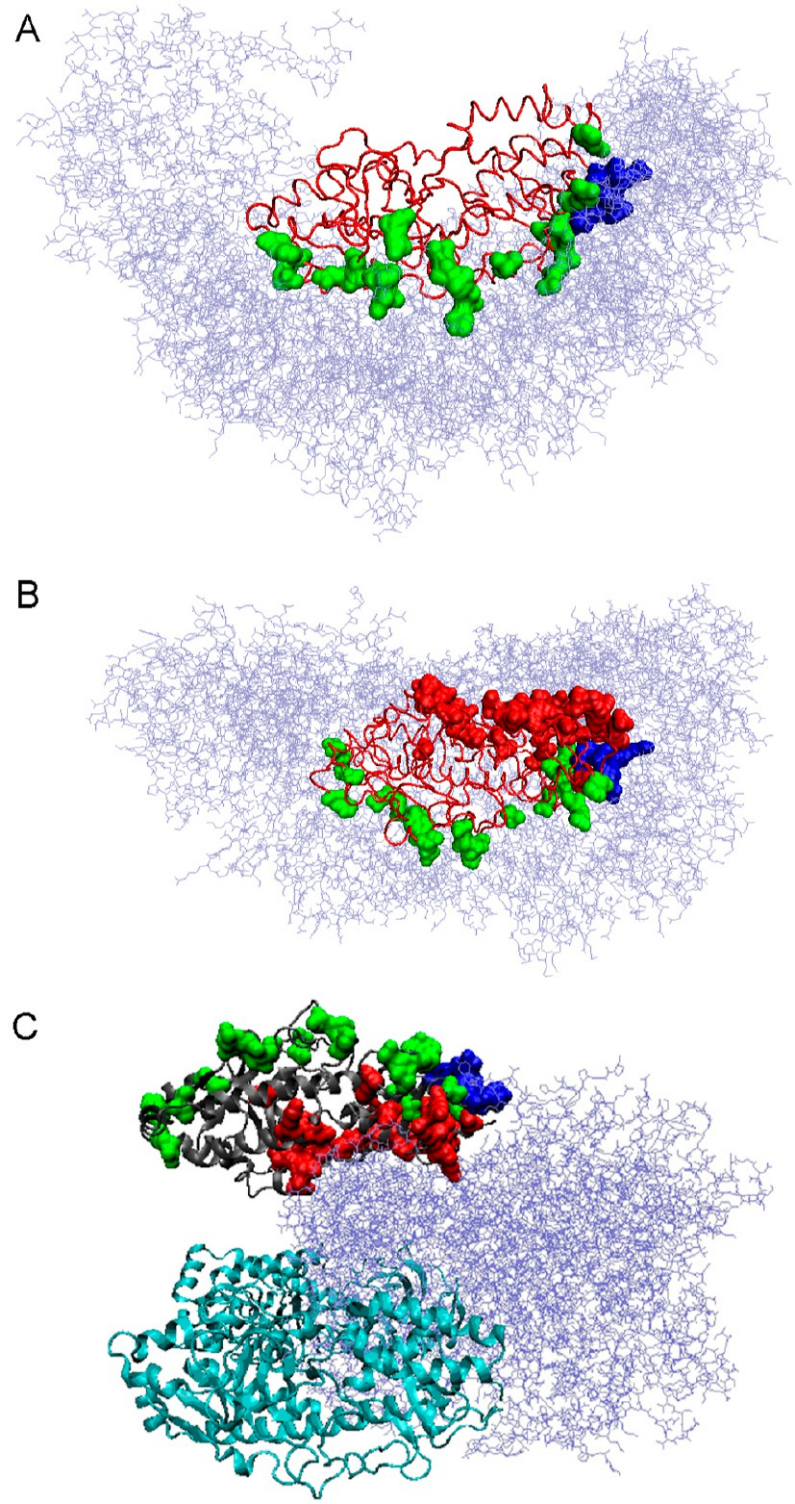
3D presentation of the reovirus mu1/σ3 complex structures. (A) Q chain (red line) in complex with the chaperonin fragment (ice blue, chains). Green residues (space-filling representation) are those interacting with chaperonin, and dark blue residues (space-filling representation) are those interacting with chaperonin and later with the WT form of protein in the reovirus mu1/σ3 complex. (B) As in (A) with red (space-filling representation) residues interacting later with the reovirus mu1/σ3 complex. (C) Reovirus mu1/σ3 complex WT (the gray chain), G chain with green residues are those formerly interacting with chaperonin, red residues are those interacting with other chains of virus complex but not interacting with chaperonin, and dark blue residues are those interacting both in complex with chaperonin chains and now with the virus complex. H and I chains (cyan chains) are the same as the G chain.

A higher number of residues are also involved in the interchain interaction in reovirus mu1/σ3. The specificity of the interaction within TRiC and reovirus mu1/σ3 is revealed by juxtaposing three 3D structure presentations of the Q/G chain with residues involved in the interface construction (Figure 5). The locations of residues involved in the Q chain interactions with the TRiC chains are shown in Figure 5A, whereas completely different residues were involved in interactions in the reovirus mu1/σ3 complex, as shown in Figure 5B; also shown are those residues that are present at the interface in both complexes discussed (Figure 5A–C, blue). The differences in the structures of the interfaces are clearly shown in Figure 5B.

The juxtaposition of the profiles (Figure 4) with the visualized residues involved in the interaction with other chains of the corresponding complex reveals a different form of the complex structure. The visualization of these residues in the 3D representation (Figure 5A–C) suggests a directed folding process in the complex with TRiC to obtain a structure of the future interface that could interact with the reovirus mu1/σ3 complex but is not involved in interactions with TRiC.

The evaluation of the status of the residues involved in the interaction with the remaining components of the complex reveals differences in the structures of the respective interfaces (Table 2).

**Table 2:** Status of the Interface (P–P) between Individual Chains in the Respective Complexes, Expressed by the RD and *K* Parameter Values, and the Status of the Part of the Respective Chain Lacking Residues That Belong to the Interface^a^

interacting chains	P–P		No P–P	
	RD	*K*	RD	*K*
Q-TRiC (green)	0.553	0.5	0.702	1.1
G-Mu1 (red)	0.637	1.1	0.696	1.1
Q-TRiC and Mu1 (blue)	0.414	0.0	0.687	1.1
Q-TRiC or Mu1 (red + green)	0.595	0.9	0.715	1.1

a Colors identifying respective residues are given in brackets, as shown in Figures 4 and 5.

**Figure 6:**
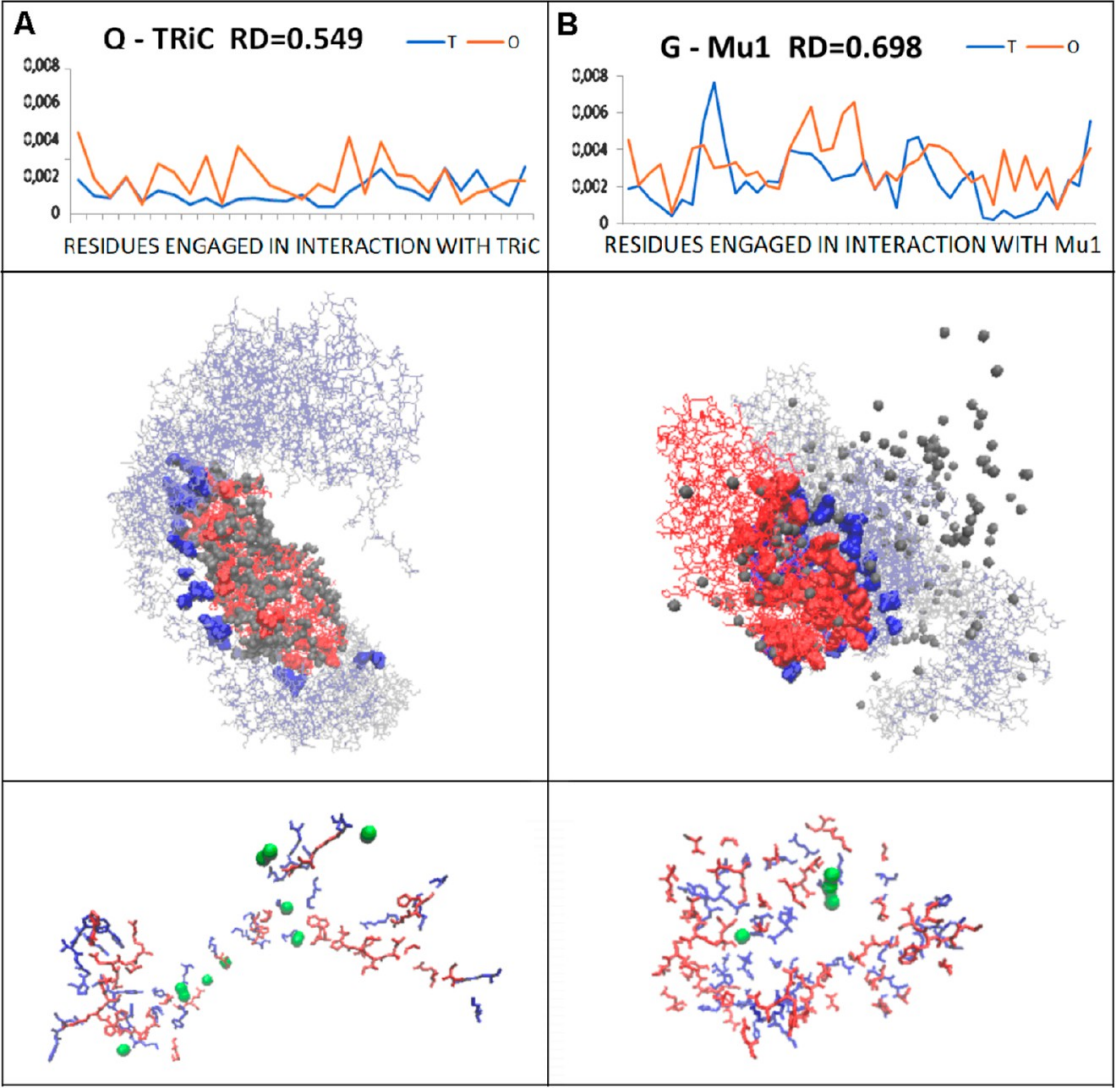
Visualization of water participation in structuralization of Q and G chains in complex with (A) TRiC and (B) Mu1, respectively; *T* and *O* profiles for residues engaged in contacts with other chains in complex with TRiC and Mu1, respectively. 3D structures of chain Q-TRiC and G-Mu1 complex, respectively. Gray balls are water molecules, red balls are residues interacting with other chains, red lines represent the remaining part of chains, and blue balls are residues of target chains The lowest row—residues participating in interface construction (blue—target chains, red—Q and G chains, respectively) with water molecules mediating the interchain interactions.

The RD and *K* parameter values determined for the residues (components of the interface) and for the remaining part of the complex reveal the low contribution of the interface itself as a factor influencing the structuring. This is indicated by the RD and *K* value s determined for structures lacking the residues that constitute the corresponding interfaces (Table 2, column no P–P). The RD values were similar, and the *K* value s were the same for all the cases discussed. The G-Mu1 (red) interface shows a status comparable with that of the part lacking this fragment. This means that the interface was not constructed in a way that would distinguish this section from the rest of the complex. The Q-TRiC and Mu1 interfaces (a fragment involved in both the Q and G chains, blue) show low RD values and *K* = 0.0, which indicates local entry into the micelle-like structuring. As this is a surface zone, this means that polar residues are involved in the interface structure. Excess hydrophobicity is evident in the profiles (Figure 6), although in the present analysis, this appears to be negligible from the perspective of the overall structure of the complex. The status of the interface within the Q chain (and thus complexation with TRiC) appears to be better suited to the micelle-like arrangement, but this is a state far from this type of ordering. The RD value of residues interacting with TRiC was 0.549 and that of residues interacting with Mu1 was 0.698.

The analysis of the water participation is shown in Figure 6. The active participation of water molecules in the Q-TRiC complex is highly specific with high packing around the folding chain Q. The distribution of water molecules in the G-Mu1 complex represents the distribution at a certain distance from the protein. However, some water molecules are located close to the G chain (Figure 6B). The status of interface residues in complexes differs when expressed by RD values (calculated solely for residues engaged in interaction in chains Q and G, respectively). The higher RD values for the G-Mu1 complex suggest a higher preparation to participate in the reovirus complex (Figure 6A,B). This local disorder from the structural perspective of individual chains, as explained using the FOD-M model as the criterion, is prepared by structuralization in the TRiC-Q complex. The interface in the Mu1 complex engages more residues distributed on a specific surface (44 amino acids), whereas residues engaged in the interface in the TRiC-Q complex engaged creates the linear arch construction (29 amino acids).

Thus, the juxtaposition of the structures (Figure 5) reveals the purpose of the involvement of residues in the interaction with TRiC to prepare an appropriate surface for the future complexation of the native form of the final reovirus σ3 capsid structure. The location of part of the chain involved in complexation within both complexes (navy blue) is notable. This section acts as an “anchor” common to both mechanisms, orienting the whole chain adequately to the final structure within the complex.

**Figure 7:**
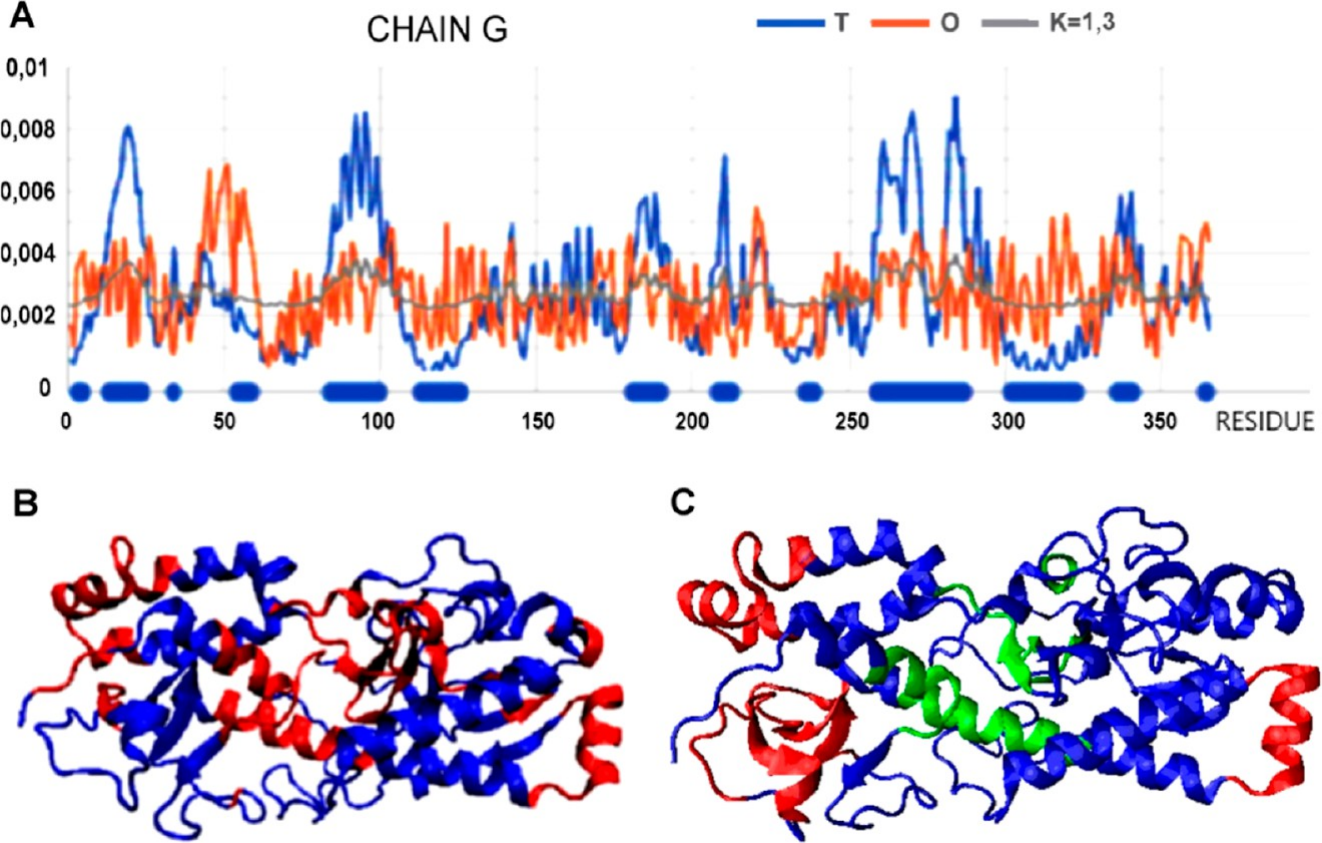
Characterization of the Q/G chain (the *x*-axis is hydrophobicity). (A) *T*, *O*, and *M* profiles with highlighted residues (solid blue circles), the elimination of which results in an RD < 0.5. (B) 3D presentation with highlighted residues, the elimination of which results in an RD < 0.5, as indicated in (A). (C) 3D presentation with residues showing local excess hydrophobicity (red) at (39–64, 69–79, 113–126, and 301–322) and local deficit (green) at (13–22, 86–101, 187–190, 259–269, and 281–287).

For a complete analysis of the structure specificity of the Q/G chain (treated jointly because of high structural similarity), assessing the degree of mismatch of the *O* distribution to the *T* distribution identified residues showing significant differences in *T*_i_ and *O*_i_ levels (Figure 7A). Their location is visualized in Figure 7B. The residues were identified by eliminating those from all the amino acids present in the chains, leading to RD values < 0.5. These positions are also highlighted in the profiles (Figure 7A).

In addition, the location of residues showing local excess (red) and deficit (green) hydrophobicity (Figure 7C) determined based on the *T*, *O*, and *M* profiles was indicated.

Figure 7C shows the variation in the status of individual chain fragments, distinguishing between positions with a local excess of hydrophobicity (red) and a local deficit of hydrophobicity (green). The presence of relatively long sections showing deviations from the micelle-like distribution reveals an external force-field effect.

### 3.2 Respective Contribution of the Q and G Chains to the Structure of the Complexes

The aim of the analysis presented here is to determine the role of the external force field in the folding process. Therefore, the focus in this section is on the Q chain TRiC structure.

The set of the *T*, *O*, and *M* profiles for the Q chain treated as a part of the TRiC complex (3D Gaussian function spanning the full complex, a fragment of profiles describing the status of the Q chain) reveals an similar? even within the *O* profile against a significant variation in the expected hydrophobicity level (*T* profile). The high *T*_i_ values for the Q chain result from its location in the central part of the complex, where a high level of hydrophobicity is expected. The *O*_i_ values, however, appear to oscillate around a constant level that is much lower than the expected values. The *M* profile is distinctive as this takes the form of a straight line parallel to the *X*-axis, indicating a significant approximation of the *M* distribution to the *R* distribution. The *R* distribution represents a system completely devoid of variation at the hydrophobicity level. This implies no effect of polar water that directs the centralization of hydrophobicity within a molecule or complex. This status is also expressed by the high value of the parameter *K* = 3.8, which indicates a high proportion of the opposite or complemented Gaussian function [*T*_MAX_ – *T*_i_]_*n*_. This operation is explained in Figure 2. This high value of *K* may be interpreted as overestimated due to the absence of a hydrophobic core in the central part of the construction.

**Figure 8:**
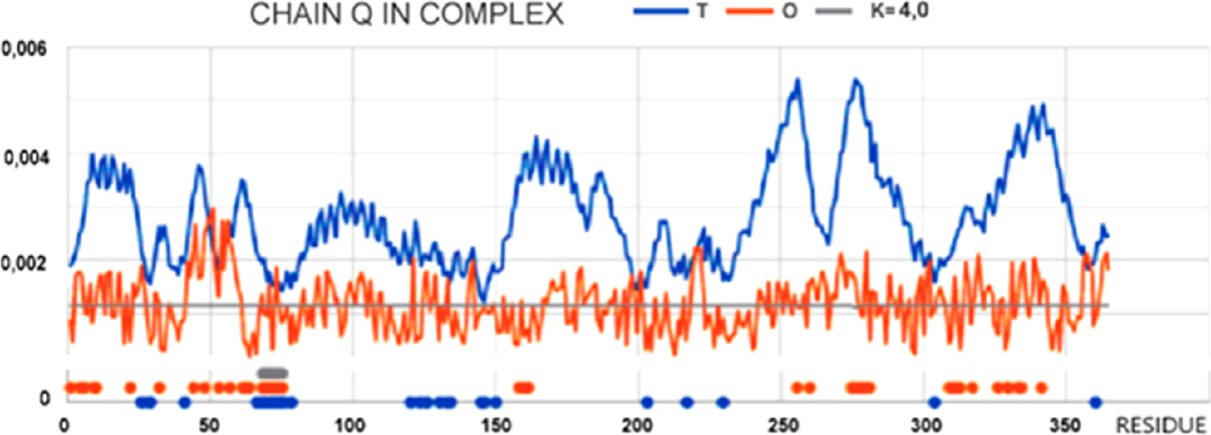
Set of *T*, *O*, and *M* (vertical axis: hydrophobicity) profiles to determine the status of the Q chain as a component of the TRiC complex.

Such a state can be described as a hydrophobic milieu or a nonpolar milieu, where no influence of the aqueous environment directing toward the micelle-like ordering is evident. The juxtaposition of the *O* and *M* profiles (Figure 8) indicates a reproduction of the hydrophobicity distribution (*O*_i_) imposed by the TRiC-derived field (*M*_i_). The status of the Q chain within the complex clearly ignores the expectations expressed by the *T*_i_ profile.

### 3.3 Specificity Expressed by the External Force Field Created by TRiC

A special object of analysis is the TRiC complex itself as the provider of the external force field for the folding Q chain. The high RD and *K* value s for the capsid were due to reasons, such as the free space in the central part of the capsid. The status of individual chains as components of the capsid and treated as individual structural units are given in Table 3, which reveals a high degree of mismatch to the micelle-like structuring, which is comparable with the status of the entire capsid. The structure (within the meaning of the FOD-M model) is described by the high RD and, especially, *K* value s.

**Table 3:** Values Are Given in Pairs for the Corresponding Chains Remaining in the Symmetry Relation

	TRiC-X–complex (chains A–P)		individual chains	
chain	RD	*K*	RD	*K*
TRiC (A–P)	0.752	3.6		
TRiC-A/TRiC-I	0.755/0.751	3.5/3.2	0.638/0.644	0.8/0.8
TRiC-B/TRiC-J	0.769/0.768	3.5/3.4	0.682/0.681	1.0/0.8
TRiC-C/TRiC-K	0.737/0.735	2.8/2.7	0.654/0.648	0.8/0.7
TRiC-D/TRiC-L	0.752/0.754	3.9/3.9	0.561/0.567	0.5 /0.5
TRiC-E/TRiC-M	0.777/0.777	3.5/3.6	0.652/0.644	0.8 /0.8
TRiC-F/TRiC-N	0.736/0.736	2.8/2.9	0.674/0.681	0.9 /0.9
TRiC-G/TRiC-O	0.736/0.736	2.7/2.7	0.624/0.620	0.7 /0.7
TRiC-H/TRiC-P	0.751/0.752	3.6/3.6	0.637/0.639	0.8/0.8

**Figure 9:**
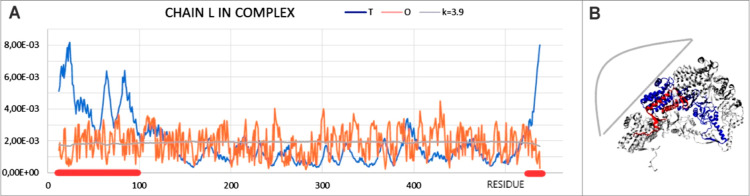
Characterization of the L chain as a component of TRiC (vertical axis: hydrophobicity). (A) *T*, *O*, and *M* profiles. Red thick line are sections with a status clearly mismatched to the expectation according to the *T* distribution, visualized in the 3D structure (Figure 9B). (B) 3D presentation of the L chain (dark blue) surrounded by the M and K chains (gray). Highlighted red sections are sections facing the inside of the chamber, which are highlighted in (A). The gray shape visualizes the location of the second symmetrical part (second domain).

The example distribution of *T*, *O*, and *R* for the selected L chain as a component of the complex (Figure 9) shows a clear divergence of the *O* distribution from the *T* distribution and a large approximation of the *R* distribution. A clear mismatch is observed for the N- and C-terminal sections. As these face toward the central part of the inside of the system, they should (as expected) show higher levels of hydrophobicity. In fact, they continue a steady distribution oscillating around the level indicated by the *M* distribution. These sections are highlighted both in the profiles (Figure 9A) and in the 3D presentation (Figure 9B).

Notably, the juxtaposed profiles represent a fragment of the profiles obtained for the entire TRiC structure. All of the other chains show a comparable arrangement. An analysis of the D chain component of the complex, given using profiles and in the 3D presentation, characterizes the structural specificity of the components of the TRiC complex.

**Figure 10:**
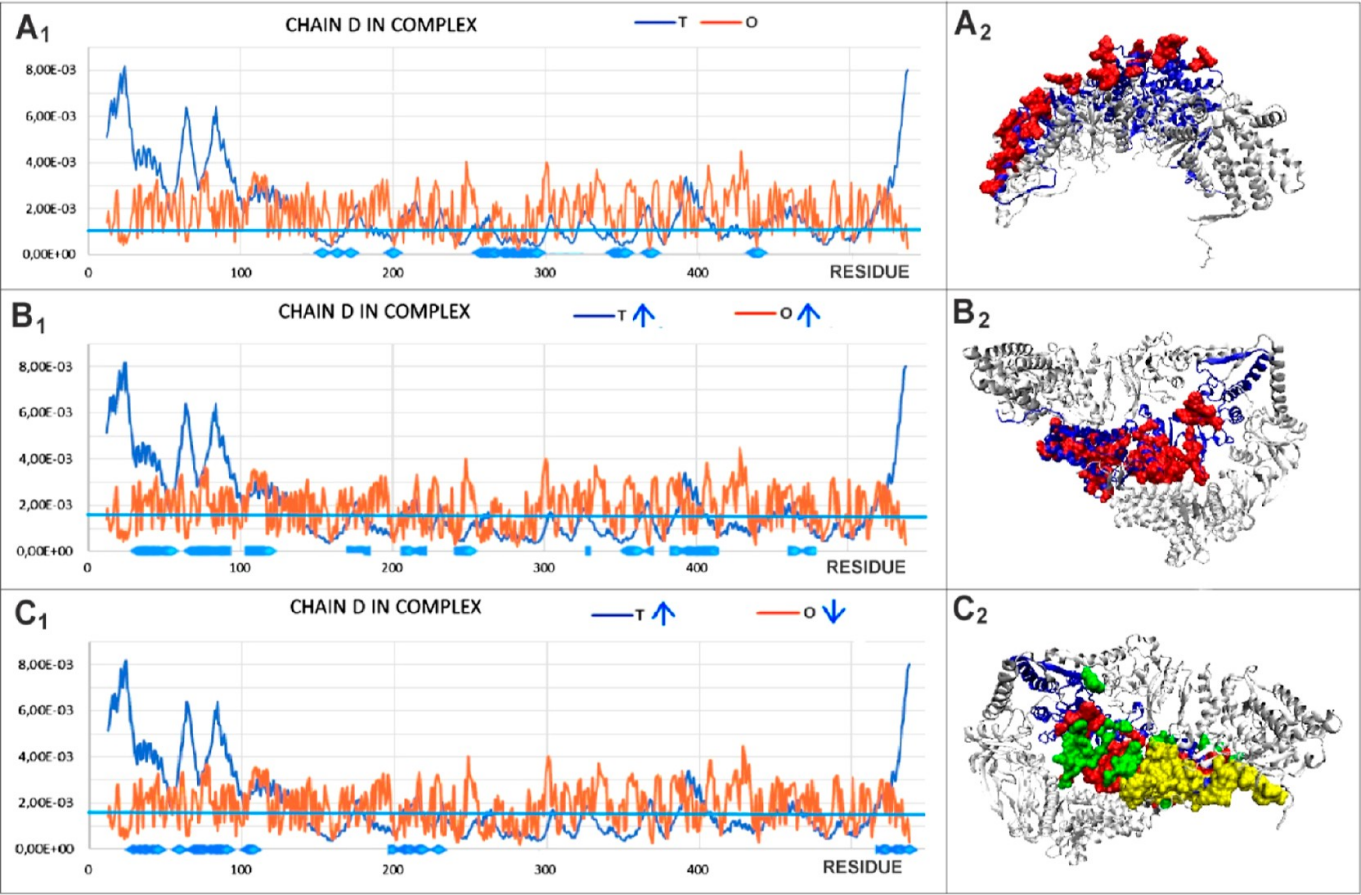
Characteristics representing the status of the D chain (dark blue) as a component of the TRiC structure. (A) Residues with *T*_i_ < 0.001 and *O*_i_ < 0.001. The blue line is the cutoff level and their location in the spatial structure. (B) *O*_i_ > 0.0015 and *T*_i_ > 0.0015. The blue line indicates the cutoff line at 0.0015. Arrows next to T and O indicate a qualification criterion (upward arrow for above, downward arrow for below) for the residues in the 3D figure on the right. (C) *O*_i_ < 0.0015, *T*_i_ > 0.0015. Green represents the inner polar inner surface, and red represents residues as in B yellow section (section 12–100) that generally show a level far below the expected one. Arrows next to T and O indicate a qualification criterion (upward arrow for above, downward arrow for below) for residues in the third figure on the right.

Figure 10A visualizes the locations of those residues meeting the condition identifying the surface location (*T*_i_ < 0.001). Fulfilling the concurrent condition *O*_i_ < 0.001 indicates the status of polar residues exposed on the surface as expected from the *T* distribution. Figure 10B allows for the location of residues meeting the condition *T*_i_ > 0.0015 and *O*_i_ > 0.0015. This implies the location of residues in the complex wall layer that is next to the surface layer. These residues can be treated as showing a hydrophobicity distribution consistent with expectations as being located slightly deeper below the surface. This layer represents a status compliant with the expectations of the model.

Residues satisfying the condition *T*_i_ > 0.0015 and *O*_i_ < 0.0015 were those where the status, contrary to expectations, indicates a hydrophobicity deficit. As shown in Figure 10C, these residues are located on the inner side of the layer constituting the walls of the system. This means that the inner surface is also covered with polar residues. Adding the N- and C-terminal residues to this presentation results in this deficit being applied to almost all of the inner surface of the TRiC system.

While the outer surface appears to represent a state comparable to the expected one (low *T*_i_ and low *O*_i_), the inner surface of the specific capsid membrane in the case of TRiC is also polar, showing the *O*_i_ levels to be lower than the *T*_i_ levels (Figure 10C). The zone visualized in Figure 10B can be interpreted as a stabilizing factor against the surrounding aqueous environment. Indeed, a slight increase in the hydrophobicity level toward the inside of the structure is visible, as expected by the surrounding water external polar field. Thus, the presence of an external polar surface of TRiC with a narrow subsurface layer, with a distribution meeting the expectation and lower-than-expected hydrophobicity levels at the polar internal surface of the chambers.

**Figure 11:**
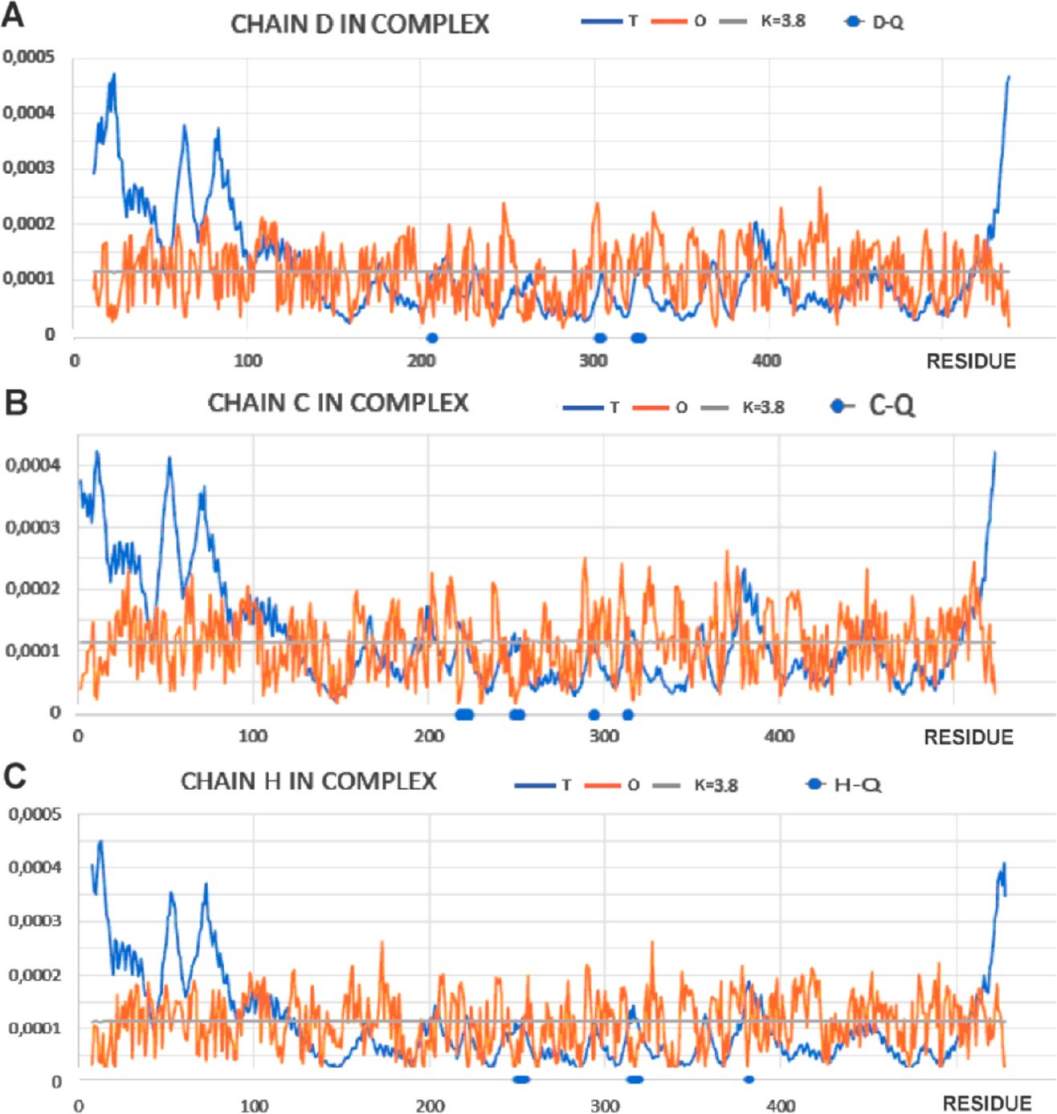
Juxtaposition of the *T*, *O*, and *M* (vertical axis: hydrophobicity) profiles of the TRiC chains involved in complexation of the reovirus σ3 capsid protein with the residues, highlighted on the horizontal axes, involved in interactions with the reovirus σ3 capsid protein. Profiles were determined for the corresponding chains treated as components of the capsid complex (TRiC). (A) chain D; (B) chain C; and (C) chain H.

An analysis of the *T*, *O*, and *M* profiles for the D, C, and H chains (chains in TRiC) interacting with the Q chain demonstrates a high similarity compared with the distributions given for the L chain shown earlier (Figure 11) despite the slightly different values of the RD and *K* parameters (Table 3). A form of the *M* distribution, common for all of the chains, is visible. The undifferentiated status of residues involved in interactions with the reovirus σ3 capsid chain is also apparent. This is mainly due to the almost exclusively polar nature of the interaction between the reovirus σ3 capsid chain and the TRiC chains.

### 3.4 Proposed Model for Chain Folding with the Components of TRiC

The detailed characteristics of chains in the reovirus mu1/σ3 complex are presented to discuss their status as individual structural units and in the complex (Table S1). High values of RD and *K* for both structural forms, especially those describing the status of individual structural units, suggest the folding process that could occur in an aqueous environment. High participation of the nonaqueous environment is necessary to obtain the forms present in these chains.

The construction of a highly complex structural form of chaperonin appears possible as a result of individual chains folding with the participation of water molecules. The interpretation of the *T*, *O*, and *M* profiles to describe the status of the M chain as part of the complex (Figure S1A) and as an individual structural unit (Figure S1B) reveals a relatively high accordance of *T* and *O* distribution in the M chain when treated as an individual structural unit. Table S1 describes the status of the chains in the complex. The analysis is also focused on P–P contacts in respect to the preparation for this type of interaction in the structure of individual chains. Such exposure is treated as preparation for the interchain P–P interaction. Table S2 also identifies the residues that represent discordance with respect to FOD organization. The identification of them is possible based on the *T*_i_, *O*_i_, and *M*_i_ profiles calculated for individual chains (Figure S1). The localization of these residues is shown in Figure S2A. Elimination of residues to lower the RD < 0.5 (Figure S2B) is often because these represent local hydrophobicity exposure. Thus, folding of an individual chain is possible because of the contribution of water molecules.

The problem of protein folding remains unsolved despite many attempts.^35^ Although increasingly effective methods are available, including AlphaFold,^36,37^ the answer to the question “Why do they fold the way they do?” is yet to be answered. The starting point for the analysis of the influence of the environment on this process is the observation that the most successful programs (models) provide, in the CASP project, correct results for a specific group of proteins but fail to account for other proteins. This gives rise to the conclusion that the proteins themselves differ so significantly that the common model (common force field) used produces highly variable results. The current analysis focuses on determining the role of the environment that affects protein folding, especially for chaperonin-mediated folding.^38^ The starting point is proteins that when folded in an aqueous environment exhibit structuring with a well-defined hydrophobic core with a polar surface (downhill, fast-folding, ultrafast-folding, and antifreeze proteins type II^18^). These groups of proteins are examples with structures expressed by low parameter values based on the FOD model: RD and *K*. Proteins described as *K* = 0.0 were identified. Single-chain enzymes show structuring with an RD just above the threshold value of 0.5. The elimination of a few residues produces significant differences in the level of *O*_i_ toward *T*_i_ and helps identify that part of the protein where the hydrophobicity pattern is micelle-like. These eliminated residues (with a local deficit in hydrophobicity due to the frequently present cavity) are generally residues that constitute the active center.^39^ The local superficial excess of hydrophobicity is often used for the complexation of another chain with a similar hydrophobicity exposure.^23^ The amphipathic membrane environment requires a hydrophobicity ordering that opposes the micelle-like ordering. This is because the surface exposure of hydrophobic residues that stabilize the protein in the cell membrane is expected.^40–43^ Here, the range of values for the *K* parameter was close to *K* = 1.0. Proteins with *K* > 1.0 require other chaperones or chaperonins to support the folding process.

The membrane and chaperones are regarded as different forms of external force fields, which, by participating actively in the folding process, guarantee a structure that is different from the one that would be imposed by the aqueous environment. Chaperones providing an appropriate external force field themselves exhibit a structure expressed with even higher *K* value s (even close to *K* = 4.0), as shown in the example of GroEL discussed here.

The aim of this analysis was to derive a mathematical representation of the external force field as a continuum written in a form expressing the *M* distribution. A simulation of the folding process with *in silico* techniques in the M field environment is expected to produce a structure obtained by protein folding in an environment that differs from an aqueous environment. This variation in how the external force field is expressed, and thus in the orientation of the folding process, will explain the variation of results in the CASP project, where participants use somewhat “averaged” fields that do not consider environmental conditions.

A membrane protein represents a hydrophobicity distribution that differs from that of a water-soluble protein, and the structural formation of such proteins is dependent on the environment. The appropriate exposure of hydrophobic residues and, in a further step, the specific distribution of hydrophobicity within the protein are closely related to biological activity and, when interacting with the environment, including potential molecules, such as substrates. The importance of the interaction of water molecules with a diverse environment is the object of analysis in numerous studies.^40–43^ The presence of the water–air interphase in the shaking procedure, where the size of the air–water contact area is significantly increased, postulated in works on amyloid^44^ structuring, has been confirmed.^40–43^ Water structuring has also been shown to influence the stabilization of intermediates in chemical processes and probably on intermediates in the protein-folding process.^41,42^

These reports on the importance of the variation of the water molecule status^40–43^ do not exhaust the full spectrum of external conditions for the protein-folding process, which provides, in addition to structure, the biological function of proteins.^28^ Therefore, the need to use a chaperonin-type structure (here TRiC) to generate a type of external field with a specificity other than water complements the wide range of diverse environments in the form of suitable water molecules. Recording the external force field as a function expressing its specificity in the form of continuum is, as expected, relevant for understanding the highly differentiated folding process, depending on environmental conditions, including amyloids.^44–46^ A function that expresses the continuum seems to solve the problem of representing the aqueous environment by using a mathematical function, replacing the previous form of this environment that was expressed as a corresponding number of water molecules. The influence of water at the level of its individual atoms with the individual atoms of the polypeptide chain expresses the influence of water in an extremely localized and individualized manner.^40–43^ This is especially important for the structuring of water molecules when in contact with oil.^44–46^

### 3.5 Reference Proteins

Protein folding with the assistance of chaperonin is quite a complex problem. This is why the reference proteins of simpler construction are presented to make possible comparable analysis.

The set of proteins (Table 4) is selected to present gradually increased RD and *K* value s.

**Table 4:** List of Proteins Analyzed in This Part of the Paper^a^

PDB ID	chain/fragment	characteristics	RD	*K*	ref
4MCX	A (192–343)	antitoxin HigA	0.387	0.1	(46)
4ABL	A	transferase	0.441	0.3	(47)
1KUF	A	hydrolase	0.412	0.3	(48)
3OQC	A (250–461)	hydrolase	0.455	0.4	(49)
1V4V	B (192–343)	isomerase	0.397	0.5	(50)
4AV3	A	hydrolase-membrane	0.703	1.0	(51)
2K4T	A	membrane prot.-apoptosis	0.603	1.1	(52)
7VWX	a	ribulose bisphosphate carboxylase	0.657	0.9	(53)
7VWX	ALL	chaperonin + chain a	0.821	6.8	(53)
7VWX	NO a	chaperonin	0.811	6.0	(53)

a The short characteristics, RD, and *K* value s are given.

**Figure 12:**
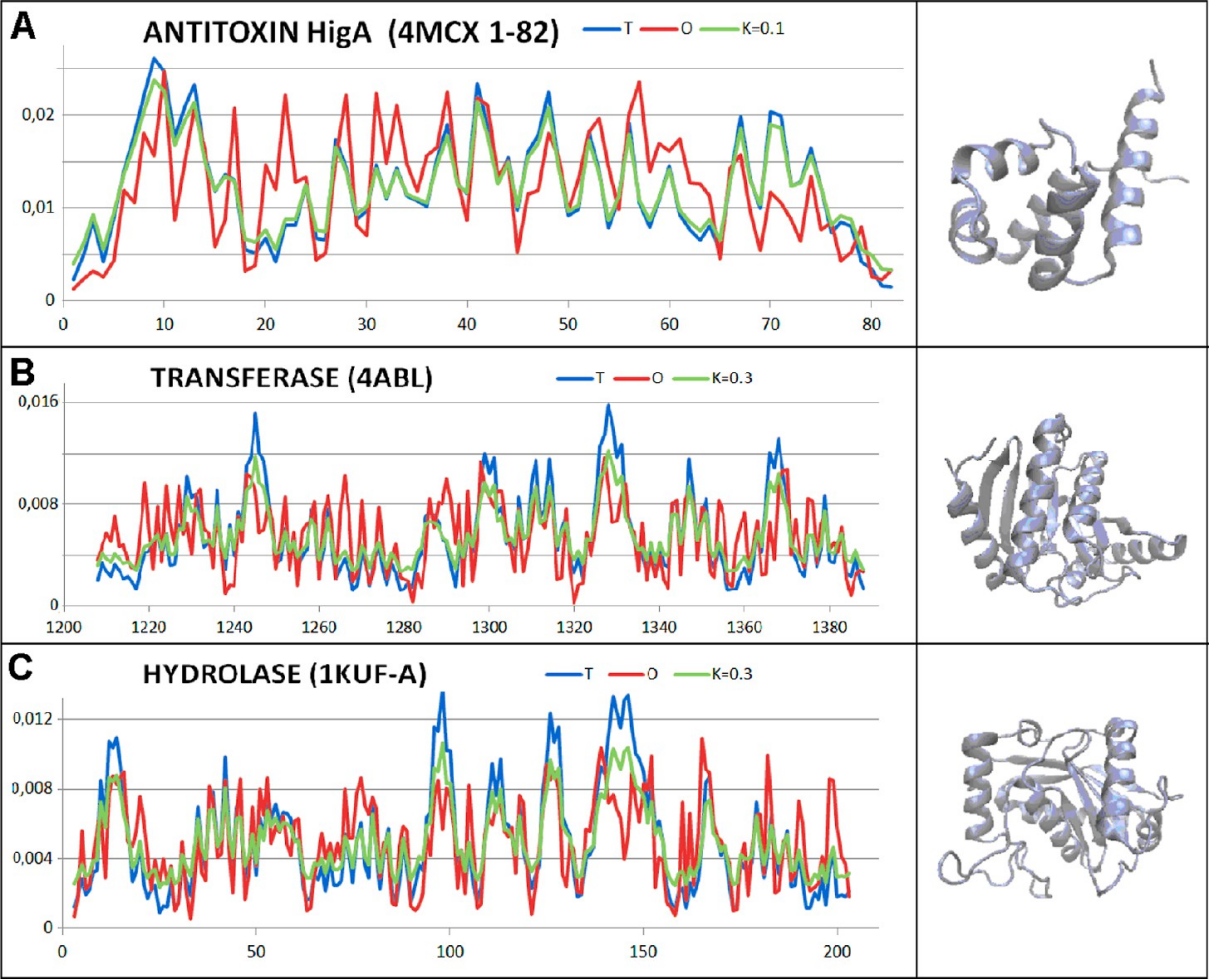
Profiles: *T* (blue), *O* (red), and *M* (green) for *K* value s for proteins of status expressed by low *K* value s together with 3D structures: (A) antitoxin HigA—domain: 1–82 residues (4MCX), (B) transferase—(4ABL), and (C) hydrolase—1KUF.

The *T*, *O*, and *M* for the appropriate *K* value are shown together with 3D presentation to visualize the different status of selected proteins. The examples of proteins characterized by low RD and *K* are shown in Figure 12.

Two enzymes with low and higher RD and *K* represent the large set of enzymes presented in.^54^

Transmembrane proteins due to hydrophobic contacts with the membrane represent higher values of RD and *K*, which is demonstrated in 13Figure 14.

**Figure 13:**
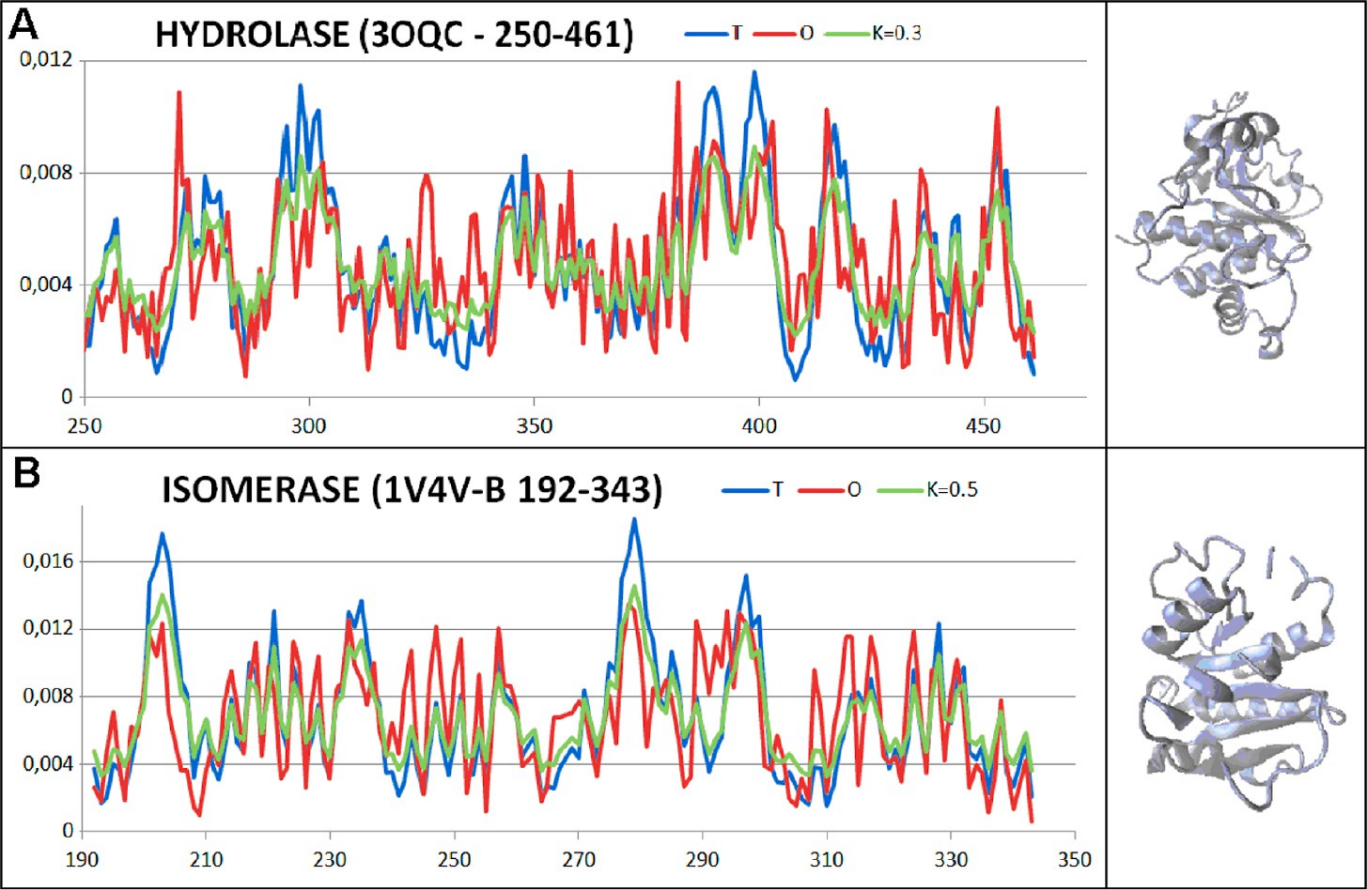
Profiles: *T* (blue), *O* (red), and *M* (green) for *K* value s for proteins of status expressed by *K* = 0.4 and *K* = 0.5 together with 3D structures. (A) Hydrolase—3OQC—domain residues 250–461. (B) Isomerase—1V4V chain B domain 192–343.

**Figure 14:**
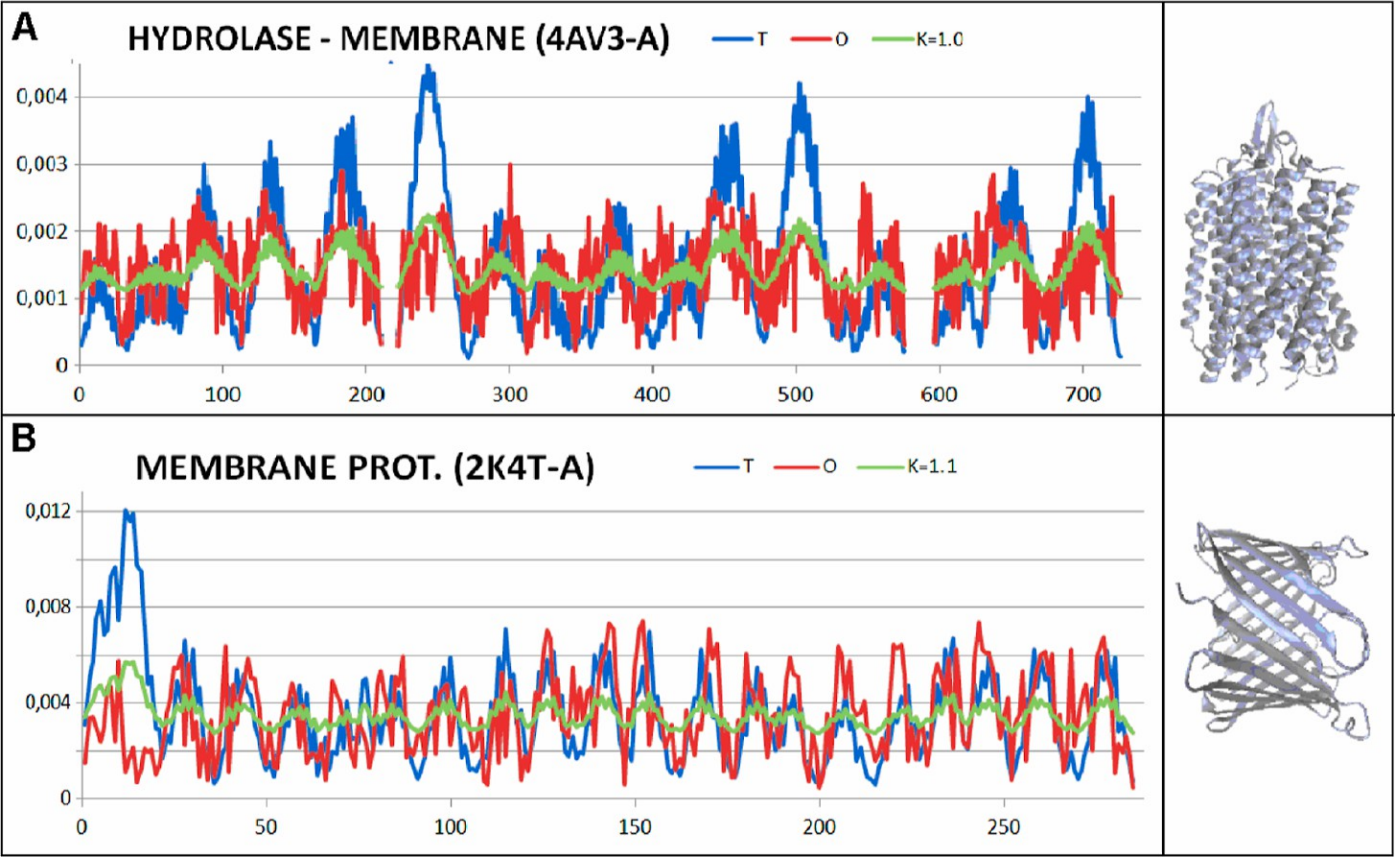
Profiles: *T* (blue), *O* (red), and *M* (green) for *K* value s for membrane proteins of status expressed by *K* ≈ 1.0 together with 3D structures. (A) Hydrolase chain A (4AV3). (B) Membrane protein—chain A (2K4T).

**Figure 15:**
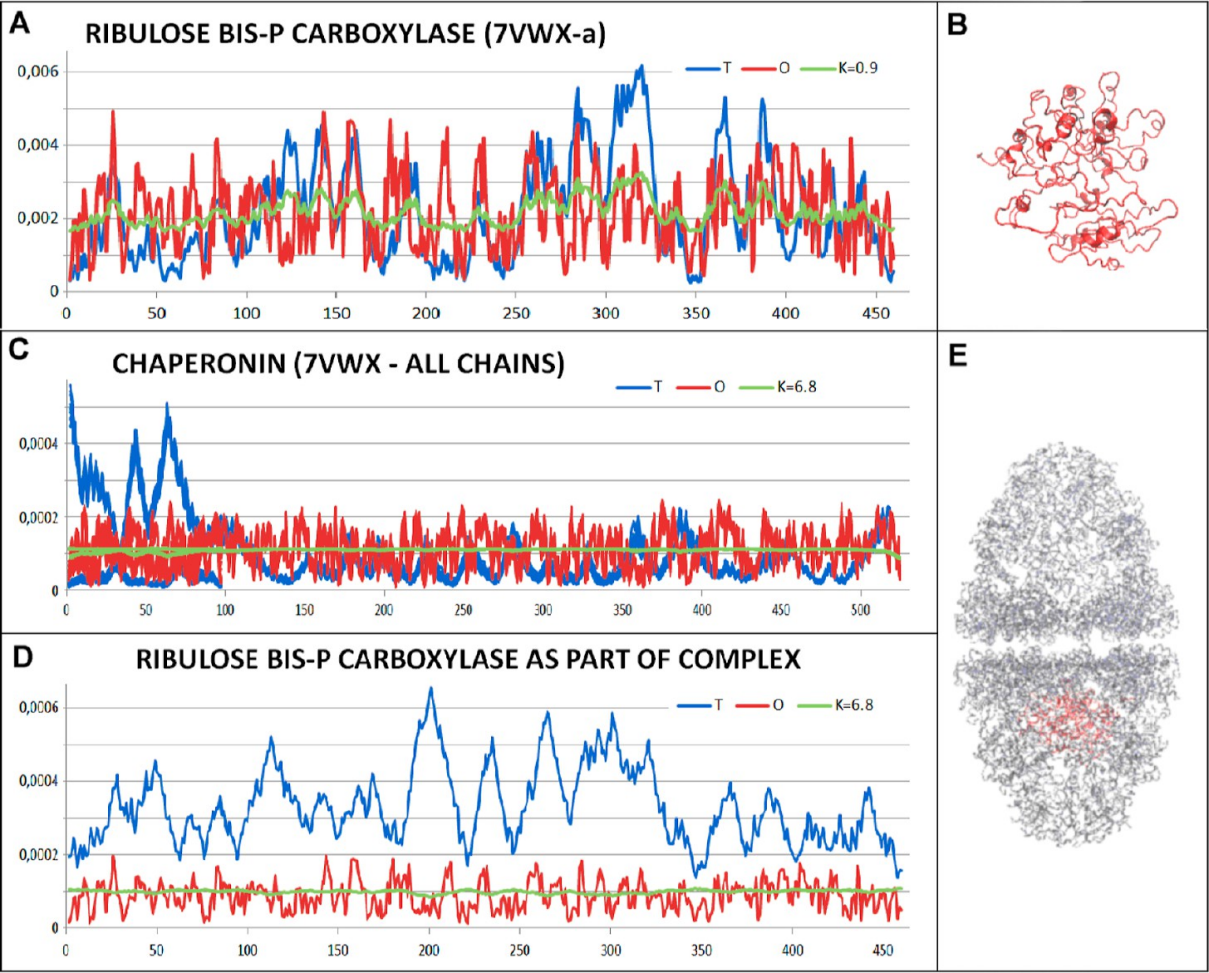
Profiles: *T* (blue), *O* (red), and *M* (green) for *K* value s for the chaperonin system together with 3D structures. (A) Folding protein ribulose bisphosphate carboxylase—structure as it is present in complex. (B) 3D structure of ribulose bisphosphate carboxylase as it appears in complex with chaperonin. (C) Profiles: *T* (blue), *O* (red), and *M* (green) to characterize the chaperonin (all chains overlapped). (D) Profiles: *T* (blue), *O* (red), and *M* (green) to characterize the ribulose bisphosphate as part of the complex. (E) Complete chaperonin together with the folding protein ribulose bisphosphate carboxylase (red).

The reference proteins represent the examples of differentiated status as expressed by the FOD-M model. The list is presented in the form of gradually increased parameter values to make possible assessment of the special status of discussed proteins folded with the assistance of supporting proteins. The example of another protein (ribulose bisphosphate carboxylase) is shown to visualize the significant influence of chaperonin on the folding process. It suggests the universal character of the model applied to characterize the specificity of the folding system with the chaperonin participation (Figures 13 and 15).

## 4 Conclusions

The conclusions of this study are based on the present work supplemented by the results of analyses of several proteins of varying status in an assessment based on the FOD model.^14,15,27–32,38,44–47^ The structure of the protein, a component of reovirus mu1/σ3, has a hydrophobicity distribution that is far from the micelle-like arrangement (centrally located hydrophobic core with a polar surface). The arrangement in this protein, characterized by a high degree of hydrophobicity dispersion with a high degree of mismatch with the micelle-like arrangement, suggests the involvement of a specific nonaqueous environment that is actively involved in the folding process. The presence of the GroEL chaperonin provides a specific external force field, which, because of the high value of the *K* parameter (*K* > 4), creates conditions with water molecules present; however, its structuralization is different with respect to external water conditions. This is why the hydrophobicity distribution appears rather to correspond to a uniform distribution of hydrophobicity across the entire structure of the complex.

The specificity of protein structuring (folding in the presence and with the participation of chaperonins) is an example of the effect of the external force-field presence, which produces a final protein structure status that is far from the micelle-like arrangement.^28^ The example discussed represents a component of a certain set of proteins (prefoldin,^3^ chaperone^37^) that provide a specific external force field, which, by active participation, affects the structuring of the final form of the protein. The evaluation and classification of these diverse external force fields also apply to the aqueous environment (that provides structuring with the micelle-like arrangement^28^) and the hydrophobic environment of the cell membrane.^28–32^

The role of chaperonin in the folding process has been recognized and described.^47^ The interpretation presented in this study introduces the quantitative assessment of the role of an external force field. This model can be applied to any form of environment, making this model universal. The simulation of the folding process is assumed to occur for any degree of nonpolarity of the surrounding. The external force field represented by chaperonin is just one example from the large spectrum of environmental specificities expressed by the *K* value .

We hypothesize that the introduction of a mathematical description of the form of the external force field discussed herein (*M* profile) in programs oriented at the simulation of protein folding will improve the results of protein structure prediction within the ab initio technique.^55^

## Data Availability

All data can be available on request addressed to corresponding author. The program allowing calculation of RD is accessible on the GitHub platform: https://github.com/KatarzynaStapor/FODmodel and on the platform https://hphob.sano.science.

## Supporting Information Available

Structure of chains as parts of the reovirus mu1/σ3 complex and discussion on whether protein (chain Q/G) can be folded without the help of chaperonin (PDF)

## Abbreviations

FOD: fuzzy oil drop model—water environment
FOD-M: fuzzy oil drop model modified—inclusion of environment conditions
RD: relative distance
*D*_KL_: Kullback–Leibler entropy
