# Supplementary material for: External Force Field for Protein Folding in Chaperonins—Potential Application in In Silico Protein Folding
Source: ACS Omega. 2024 Apr 10;9(16):18412–28. doi: 10.1021/acsomega.4c00409 (PMC11044213; doi:10.1021/acsomega.4c00409)
Supplement: Supplementary file 1 — ao4c00409_si_001.pdf [file ao4c00409_si_001.pdf]

SUPPORTING INFORMATION

EXTERNAL FORCE FIELD FOR PROTEIN FOLDING IN CHAPERONINS – POTENTIAL APPLICATION IN *IN SILICO* PROTEIN FOLDING

Irena Roterman[1,*], Katarzyna Stapor[2] , Dawid Dułak[3] and Leszek Konieczny[4]

1 Department of Bioinformatics and Telemedicine, Jagiellonian University – Medical College Medyczna 7, 30-688 Kraków, Poland, myroterm@cyf-kr.edu.pl (I.R.)
2 Faculty of Automatic, Electronics and Computer Science, Department of Applied Informatics, Silesian University of Technology, Akademicka 16, 44-100 Gliwice, Poland, katarzyna.stapor@polsl.pl (K.S.)
3 ABB Business Services Sp. z o.o. ul Żegańska 1, 04-713 Warszawa, Poland dawid.dulak@gmail.com (D.D.)
4 Chair of Medical Biochemistry – Jagiellonian University – Medical College, Kopernika 7, 31-034 Kraków, Poland, mbkoniec@cyf-kr.edu.pl (L.K.)

Corresponding Author: Irena Roterman, myroterm@cyf-kr.edu.pl

The aim of the analysis is to assess the role of the external force field for the protein folding process; therefore, the characterisation of the reovirus σ3 complex is presented separately.

Table S1 reveals analogical values for the reovirus σ3 complex, comparable status of the chain treated as an individual structural unit extracted from both compared complexes.

The assessment of the status of the Q and G chain in the complexes already shows more significant differences suggesting the folding of the chain within TRiC as closer to the rules arising from the micelle-like model. This could be interpreted as an easier formation of the final structure as part of the complex with TRiC in order to prepare this protein for a later role as a component of the reovirus σ3 capsid complex with a status highly unsuitable for a micelle-like arrangement.

What also deserves attentions is the high K value for the entire reovirus mu1/sigma3 complex, whose status is certainly required for the viral complex to perform specific biological tasks.

In contrast, the extremely high K value for the TRiC/Q chain complex and for the A-P chain system indicates a unique form, which will be interpreted later in the present work.

	reovirus mu1/sigma3 complex		CHAINS AS INDIVIDUAL UNITS	
	RD	K	RD	K
Complex (1JMU)	0.792	1.6		
Chains – A,C,E	0.731/0.658/0.792	0.5/0.4/0.6	0.845/0.845/0.844	1.7/1.7/1.7
Chains – B,D,F	0.773/0.743/0.755	1.1/1.1/1.2	0.7760/775/0.775	1.8/1.8/1.8
Chains – **G**,H,I	0.786/0.717/0.778	1.4/1.1/1.4	**0.719/0.719/0.720**	**1.3/1.3/1.3**

Table S1. The RD and K parameter values for the structural components of the reovirus mu1/sigma3 complex. G chain highlighted in bold – the object of comparative analysis with the Q chain in the complex with TRiC.

The values of the RD and K parameters for the reovirus mu1/sigma3 complex and its components indicate the presence of structures far from those being a specific product of the influence of the aqueous environment on the structuring of the proteins in question. Indeed, all components – particularly including the G chain in question – show high K values above K=1.0. Only the A, C and E chains in the complex (as its components) show adaptation to structuring that results from the influence of the aqueous environment (relatively low K values), although the RD values are very high. Probably, these chains constitute a significant factor in the stabilisation of this complex in the aqueous environment.

A detailed analysis of the T, O and M profiles in both complexes reveals characteristic features of the Q/G chain structure. Above all, the common high RD and K values are important, although these values are slightly higher for the reovirus mu1/sigma3 complex.

1. **IS IT POSSIBLE TO FOLD THE PROTEIN (CHAIN Q/G) WITHOUT THE HELP OF CHAPERONIN?**

An answer to this question can be given through an operation based on the fuzzy oil drop model involving the elimination of residues showing the greatest differences in the Oi status against the Ti status. The gradual elimination of such residues causing a reduction in the RD values leads to the identification of that part of the protein which meets the condition of compatibility of the Oi distribution towards the Ti distribution. Achieving the value RD <0.5 in this procedure allows the identification of that part of the protein that represents a micelle-like status, which can theoretically be obtained as a result of the active presence of the aqueous environment in the folding process. Very often, few residues identified as disrupting the micelle-like arrangement are those associated with the biological activity of the protein in question. A local deficit indicates the presence of a cavity, which often turns out to be a substrate/ligand binding cavity [litS2MY]. A local excess of hydrophobicity on the surface indicates a potential complexation site for another protein with a similar enthalpically unfavourable exposure [S3litMY]. In such cases, the elimination of several residues is sufficient to obtain the RD level <0.5.

In the case of the Q/G chain, obtaining an RD value < 0.5 requires the elimination of as much as 36.2% of all residues from the chain (in this case RD = 0.497). In addition, these residues are scattered along the entire chain, making it an overall mismatched system in terms of the relationship of Oi towards Ti. It can be speculated that the presence of such numerous and scattered residues causing an incompatibility of the R and T distributions makes the pursuit of a micelle-like form as unintentional in the folding process of this chain.

Here, we can compare this with the status of enzyme lysozyme, where RD >0.5 reduces its value to RD <0.5 after removal of only three residues (including two catalytic residues) [30]. In comparison, lysozyme represents a localised incompatibility that stems from sequence specificity alone. The proposed form of axiom expressed in the traditional form "sequence determines structure and function" can be replaced by the statement "sequence determines the form of local deviation from micelle-like distribution." And this is precisely the case in enzymes exemplified by lysozyme or peptidylprolyl isomerase [20lit JCBiochemistry].

2. CHAINS IN CHAPERONIN – HOW DO THEY FOLD?

CATH does not identify domains in the single chain structure [S1]. Fig.1 shows an ordering in the form of two centres with the Oi distribution similar to the T distribution, which would suggest the presence of two subunits, although their rather close association does not allow them to be treated as independently folding units as defined by the domain definition.

This raises the question of the folding mode of TRiC itself. If it represents a system with high RD and K values, does chaperonin itself need another chaperonin to obtain a form that provides an adequate field for the folding proteins?

This question can be answered by analysing the hydrophobicity distribution for a single chain – a chaperonin component.

The T, O and M distributions for the example chain (M chain) obtained for this chain as a TRiC component show significant deviations from the reference distribution – the T distribution. The expected hydrophobicity concentration in the N-terminal section is not reproduced by the O distribution. Furthermore, the O distribution has a form very similar to that of the linear R distribution (RD = 0.777 with K = 3.6). These high values indicate significant deviations from the structuring directed by the aqueous environment. The value K = 3.6 is interpreted by the FOD-M model as an example of a very strong influence of the non-aqueous environment on the resultant structural form. A structure with the characteristics given in Fig. S1 cannot be achieved under conditions of surrounding polar water. It should be noted that the conclusions given here apply to the whole complex as the M chain considered as part of the system of the full complex represents the whole complex. Consequently, the structure with the distribution given in Fig. S1.A – (profiles) is not a “product” of the influence of the aqueous environment.

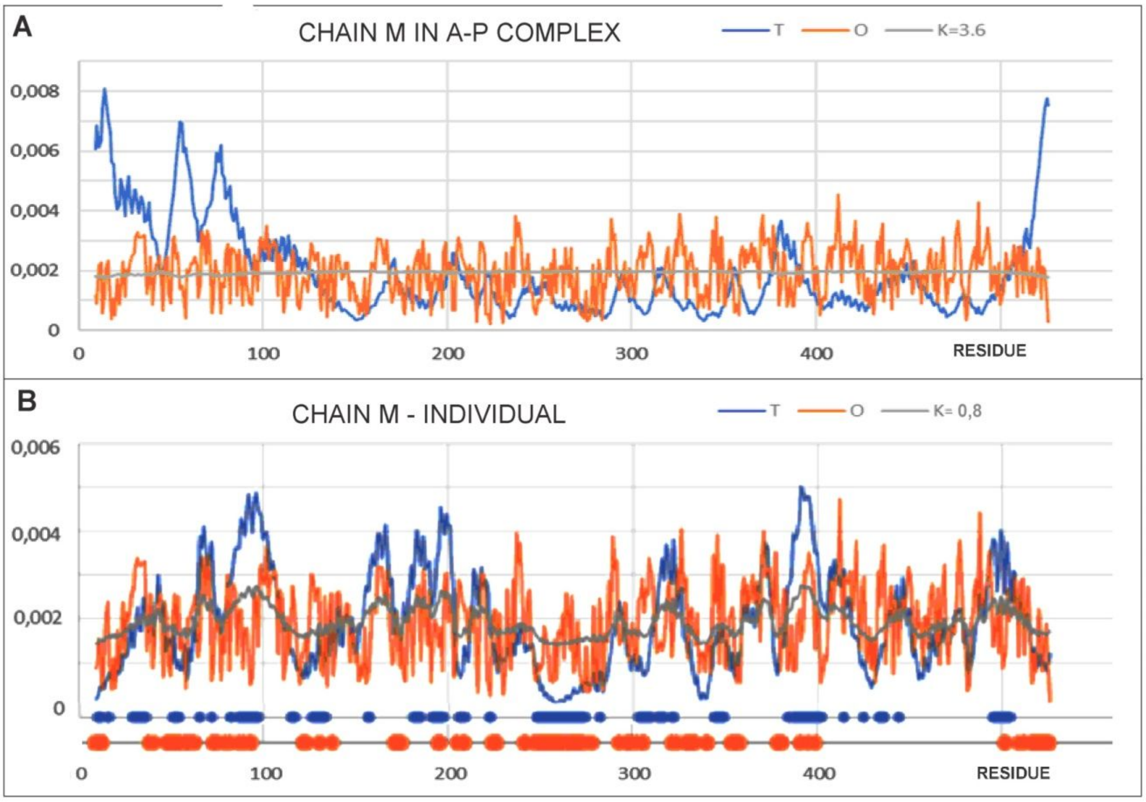

Figure S1. Set of T, O and M profiles for

A – M chain as a component of the TRiC complex – a representative of the whole system

B – M chain treated as an individual structural unit.

Dark blue dots – residues that need to be eliminated to obtain a part of the chain with RD < 0.5. Red – P-P interaction. Vertical axes represent hydrophobicity.

The interpretation of the structure of the M chain treated as an individual structural unit reveals a certain similarity of the O distribution to the T distribution with the M distribution defined by the value K=0.8. This indicates a significant approximation to the micelle-like arrangement, although it is still far from an ordering of this type. The elimination of residues showing a high deviation of the Oi value against the Ti value comprises 34.5% and this proportion is defined as significant.

The set of residues shown in Table S2 implies that residues not showing excess hydrophobicity or even showing a deficit are also involved in the inter-chain interaction. This means that other non-bonding interactions including salt bridges and hydrogen bonds are responsible for stabilisation.

In contrast, the single chain (M chain was chosen) reveals the O distribution comparable to the T distribution. The value RD = 0.652 is high, but its cause is well defined. As reported in Table S2, the considerable number of residues disrupting the compatibility of the O distribution against the T distribution is due to the local exposure of hydrophobicity, which, as also shown in Table S1, is the basis for the P-P type interaction stabilising the complex. The local exposure of hydrophobic residues is intentional in terms of preparing the protein molecule for interaction with other chains within the capsid structure.

ELIMINATED	**10-12**, 15, 16, 29-36, **51-54**, 65, 72, **82,86-97**, 115-117, 126-133, 156, 157, 181-185, **191-197**, 206, 207, **209**, 222, 223, **248-274**, 282, **303-310**, 313-317, **321,** 322, 344-349, 384, 385, **387-402**, 414, 425, 433-436, 444, **495-504**.
P-P INTERACTION	9, **11-13**, 38, 41, **48-55**, 58, 59, 61, 63, 73-75, 79, **82, 87, 90, 93, 94**, 122, 124, **131,** 138, 171-175, **195**, **196**, 205, 208, **209**, 224-227, 241, 242, 247, **249-262**, **264, 266-272**, **274**, 278, 293, 297-299, 302, **305, 306**, 321, 324, 329-334, 340, 341, 353, 355, 356, 358, 378-381, **391, 399**, **501,** 509, 512, 516-526

Table S2. List of residues disrupting the compatibility of the O distribution against the T distribution. The elimination of the listed residues results in the achievement of compatibility of the O distribution against the T distribution with RD <0.5. Bottom row – residues involved in the P-P interaction stabilising the complex. Residues present in both sets are highlighted in bold.

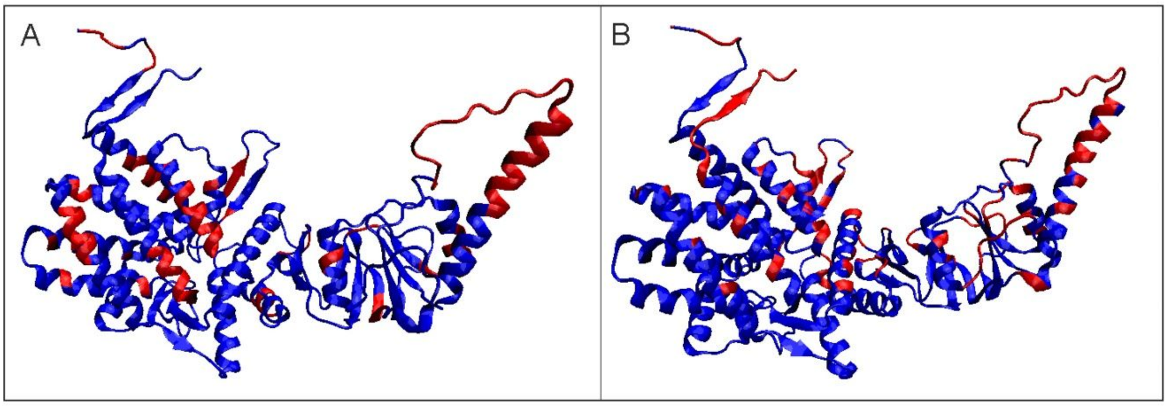

Figure S2. 3D presentation of the M chain structure

A – residues – red, whose elimination reduces RD <0.5

B – residues involved in the P-P interaction with other chaperonin chains.

In general, however, the location of residues highlighted in Table S2 and on the 3D structure in Figure S2 shows somewhat similar location.

The designation of residues with mismatched Oi status towards the Ti status occurred in the structure extracted from the complex. It cannot be assumed that this was the exact structure represented by the chain before entering the complex system. Nevertheless, the relatively high compatibility of identification of residues involved in the P-P interaction and those disrupting the FOD-compatible system seems to suggest that the mismatch in the O and T distributions is the result of meeting the requirements expected for the P-P interaction.

REFERENCES

S1. Knudsen M, Wiuf C. The **CATH** database. Hum Genomics. 2010 ; 4(3):207-12. doi: 10.1186/1479-7364-4-3-207.

S2. Banach M, Konieczny L, Roterman I. Ligand binding cavity coded in form of local defficiency of hydrophobicity. In: From globular proteins to amyloids. Ed Irena Roterman-Konieczna, Elsevier Amsterdam Netherlands, Oxford UK, Cambridge MA USA 2020. Pp. 91-95

S3. Banach M, Konieczny L, Roterman I. Protein-protein interaction encoded as an exposure of hydrophobic residues on the surface. In: From globular proteins to amyloids. Ed Irena Roterman-Konieczna, Elsevier Amsterdam Netherlands, Oxford UK, Cambridge MA USA 2020. Pp 79-90.
